# Bacteriostatic Potential of Melatonin: Therapeutic Standing and Mechanistic Insights

**DOI:** 10.3389/fimmu.2021.683879

**Published:** 2021-05-31

**Authors:** Fang He, Xiaoyan Wu, Qingzhuo Zhang, Yikun Li, Yuyi Ye, Pan Li, Shuai Chen, Yuanyi Peng, Rüdiger Hardeland, Yaoyao Xia

**Affiliations:** ^1^ College of Veterinary Medicine, Southwest University, Chongqing, China; ^2^ Guangdong Laboratory of Lingnan Modern Agriculture, Guangdong Provincial Key Laboratory of Animal Nutrition Control, Institute of Subtropical Animal Nutrition and Feed, College of Animal Science, South China Agricultural University, Guangzhou, China; ^3^ Institute of Subtropical Agriculture, Chinese Academy of Sciences, Changsha, China; ^4^ Johann Friedrich Blumenbach Institute of Zoology and Anthropology, University of Göttingen, Göttingen, Germany

**Keywords:** melatonin, bacteriostasis, MAPKs, NF-κB, ROS, inflammasome, sepsis

## Abstract

Diseases caused by pathogenic bacteria in animals (e.g., bacterial pneumonia, meningitis and sepsis) and plants (e.g., bacterial wilt, angular spot and canker) lead to high prevalence and mortality, and decomposition of plant leaves, respectively. Melatonin, an endogenous molecule, is highly pleiotropic, and accumulating evidence supports the notion that melatonin’s actions in bacterial infection deserve particular attention. Here, we summarize the antibacterial effects of melatonin *in vitro*, in animals as well as plants, and discuss the potential mechanisms. Melatonin exerts antibacterial activities not only on classic gram-negative and -positive bacteria, but also on members of other bacterial groups, such as *Mycobacterium tuberculosis*. Protective actions against bacterial infections can occur at different levels. Direct actions of melatonin may occur only at very high concentrations, which is at the borderline of practical applicability. However, various indirect functions comprise activation of hosts’ defense mechanisms or, in sepsis, attenuation of bacterially induced inflammation. In plants, its antibacterial functions involve the mitogen-activated protein kinase (MAPK) pathway; in animals, protection by melatonin against bacterially induced damage is associated with inhibition or activation of various signaling pathways, including key regulators such as NF-κB, STAT-1, Nrf2, NLRP3 inflammasome, MAPK and TLR-2/4. Moreover, melatonin can reduce formation of reactive oxygen and nitrogen species (ROS, RNS), promote detoxification and protect mitochondrial damage. Altogether, we propose that melatonin could be an effective approach against various pathogenic bacterial infections.

## Introduction

Bacteria include both pathogenic and beneficial species and are universally present in all ecosystems as colonizers of hosts (1). Bacteria can be beneficial in multiple ways, for example, *Lactobacillus* and *Bifidobacterium* species are used for yoghurt production (2, 3), *Lactococcus* species and others in cheese production (4, 5), methane producing bacteria for methane production (6), *Corynebacteria* for monosodium glutamate formation (7) and *Acetobacter* for vinegar generation (8). Bacteria, being a normal flora can also benefit hosts, such as, *Lactobacillus* species can inhibit inflammation by competing with pathogens or stimulating skin barrier recovery (9). Most, but not all bacteria present in the gut microbiome, which contains slightly more cells than a human body, can be also classified as being beneficial and relevant to health, as far as it possesses a favorable compostion (10). Pathogenic bacteria may cause mild, severe and even life-threatening diseases [e.g., septicemia (11), pneumonia (12) and meningitis (13)]. In terms of accessibility to treatments, the bacterial surface structures are of great importance. These are partially detectable by gram staining (14), according to which they are categorized as gram positive bacteria (GPB), gram negative bacteria (GNB), and others (15, 16). Examples of pathogenic GPB (PGPB) comprise *Staphylococcus aureus* (*S. aureus*) (17), *Streptococcus pneumoniae* (*S. pneumoniae*) (18) and *Bacillus anthracis* (*B. anthracis*) (19). Pathogenic GNB (PGNB) include *Escherichia coli* (*E. coli*) (20), *Helicobacter pylori* (*H. pylori*) (21), *Pseudomonas aeruginosa* (*P. aeruginosa*) (22), *Acinetobacter baumannii (A. baumannii)* (23), and *Klebsiella pneumoniae* (*K. pneumoniae*) (24). Another important pathogen, *Mycobacterium tuberculosis* (*M. tuberculosis*) (25) possesses a special waxy coating of its cell wall by mycolic acid, which largely prevents gram staining, although the cell wall structure resembles that of other GPBs and is, thus, devoid of an outer membrane (26). Also in genetic terms, *Mycobacterium* species are GPB-like, but the different surface has consequences to treatment.

Bacterial infections caused by pathogenic bacteria severely threaten public health worldwide (27, 28). Historically, the various antibiotics (e.g., penicillin, tetracycline and their derivatives as well as numerous other similarly acting compounds) usually represent the preferred armory for fighting bacterial infections (29). However, new problems (i.e., antibiotic resistance and residues) occur since the overuse and misuse of antibiotics (30–32); therefore, the development of new antibiotics that can effectively kill pathogenic bacteria and do not give rise to problematic metabolites is a matter of highest urgency. Given that very few compounds are currently under development or approval in the clinical setting, hence, repurposing compounds for novel application may become a productive alternate strategy for the combat against bacterial pathogens.

Melatonin, which shows a wide distribution within phylogenetically distant organisms from bacteria to humans (33), is synthesized from tryptophan and produced in pineal gland and in numerous other organs of vertebrates (e.g., gut, skin and bone marrow) (34–36). Melatonin is involved in many physiological processes, as summarized comprehensively (37, 38). In particular, this includes growth modulation and reproduction (39), immune regulation (40–43), anti-inflammation (44–47), antioxidative protection (48–51) and antioncogenic action (52, 53). Meanwhile, increasing evidence has accumulated for remarkable antibacterial actions of melatonin, including protection against damage by bacterial infections. For instance, melatonin has been reported to exert antibacterial effects in PGPB and PGNB *in vitro*, concerning *P. aeruginosa*, *A. baumannii* and *S. aureus* (54). Melatonin has also been shown to protect *Arabidopsis* and tobacco against *Pseudomonas syringae pv. tomato DC3000 (Pst DC3000)* (55), suggesting that melatonin reduces biotic stress by bacterial infection in plants. Also in mammals, several reports have demonstrated substantial effects of melatonin in protecting against or alleviating bacterial infections. In mice infected with *S. aureus* and *E. coli*, symptoms were strongly attenuated by melatonin (56). More impressive evidence has been obtained in septic mice treated with cecal ligation and puncture, in which the decisive effects of melatonin were related to inhibition of NO-mediated inflammation in connection with mitochondrial protection (57–59). Efficacy of melatonin was also multiply demonstrated in human sepsis, including that of neonates, which again underlines the excellent tolerability of this agent (60–62). Despite the emerging role of melatonin in fighting against bacterial infections, the protection mechanisms are only evident in the above-mentioned murine studies, plus additional anti-inflammatory effects that have been recently summarized (46, 63). Concerning protection against bacterial infections in plants, promising data have been obtained on the effects of stress-related genes and phytohormones, but still require further elucidation. Of note, with regard to these numerous highly valuable and beneficial organisms, which also represent an immunological quasi-self, one should melatonin not expect to generally act as an anti-bacterial agent. Moreover, various bacteria tested in this regard have been shown to synthesize themselves melatonin, sometimes in relevant quantities (64, 65). Among intestinal bacteria, the formation of melatonin was first described in *Escherichia coli* (66). Bacteria are even regarded as the evolutionary source of melatonin in eukaryotes, in connection with the uptake of α-proteobacteria and cyanobacteria as ancestors of mitochondria and plastids, respectively (67, 68). This has an important consequence for the consideration of melatonin as a bacteria-controlling agent. As many bacteria synthesize this compound or live, at least, in close community with melatonin-producing microbes, one cannot expect anti-bacterial effects at low doses of melatonin to which these organisms are exposed anyway under physiological conditions. However, this does not hinder bacteriostatic actions at elevated concentrations and protection of hosts from damage by bacteria or by inflammatory responses induced by bacterial challenges.

In this review, we summarize the findings related to the antibacterial actions of melatonin *in vitro* and *in vivo*. Thereafter, we detailedly discuss the potential mechanisms whereby melatonin influences pathogenic bacteria as well as their deleterious effects on hosts. These concern: 1) *in vitro*: formation and detoxification of free radicals; regulation of bacterial replication by interference with the cell wall and exhaustion of intracellular substrates and micronutrients (e.g., iron); blockade of bacterial glucose and glutamate metabolism as related to bacterial cell division; 2) in plants: upregulation of genes related to phytohormone-dependent defense signaling as well as MAPK signaling; 3) in animals: involvement of multiple signaling pathways (e.g., NF-κB, STAT1 and Nrf2) related to anti-inflammatory and antioxidant control and mitochondrial protection. The review highlights the huge potential of melatonin in counteracting the growing threat of bacterial infections.

## The Basic Physiology, Pathophysiology and Clinical Safety of Melatonin

Melatonin is a methoxyindole, mainly synthesized and secreted by the pineal gland at night under normal light and dark conditions, the main physiological functions of melatonin are related to hormonal properties (69). Melatonin transmits the information "darkness" and contributes to the synchronization of circadian oscillators (70), which is an important physiological sleep regulator in diurnal species including humans (71). In addition, melatonin is involved in numerous other physiological processes, such as regulation of blood pressure (72), glucose (73), and body temperature (74), suppression of oncogenesis (75), immune function (41), oxidative stress and inflammation (76). It is recognized that the "physiological" dose is the same as the peak plasma melatonin level at night, and the difference between the physiological and pharmacological effects of melatonin is not always clear, but is based on the consideration of the dosage rather than the duration of the hormone message (77). Indeed, the secretion of melatonin can be disturbed in the context of many pathophysiological conditions, which may increase the susceptibilities of the diseases (including infectious diseases) as well as increase the severity of symptoms or change the courses and outcomes of the diseases (78).

Of note, safety is important when melatonin is considered for clinical treatment. Clinically, 3mg, 6 mg and 10 mg melatonin showed satisfactory safety in patients (79–81). Moreover, there was no side effect of 1g/d melatonin for a month in humans (82). It has been shown that melatonin doses up to 800 mg/kg failed to cause any death in mice and it was impossible to obtain its LD50 (median lethal dose) in rats (83, 84). In study on Amyotrophic lateral sclerosis (ALS) patients, melatonin 300 mg/day was applied for 2 years and found to be safe (85). However, a trial of long-term controlled melatonin release for the treatment of sleep disorders in children with neurodevelopmental disabilities reported mild adverse effects, including seizures, cold/flu/infection, gastro-intestinal illness, agitation, anxiety and headache (86). In addition, the short- and intermediate-term administration of melatonin produced only minor adverse effects such as agitation, dizziness, headache, nausea and sleepiness in clinical studies on children; in clinical studies on adults, dizziness, paresthesias in the mouth, arms or legs, mild headaches, numbness and dyspnea aggravated; psychomotor impairment, sedation, disorientation, and amnesia in surgical patients; mild headache, increased sleepiness and skin rash in critically ill patients; daytime sleepiness in elderly (87, 88). Moreover, a recent systematic review showed that the most frequently reported melatonin adverse effects were daytime sleepiness (1.66%), dizziness (0.74%), headache (0.74%), other sleep-related adverse events (0.74%), and hypothermia (0.62%); but serious or clinical significance adverse events, including agitation, palpitations, nightmares, mood swings, fatigue, and skin irritation, were very few (89). Therefore, compared to other antibiotics, as a natural small molecule, melatonin is relatively safe with a low risk of side effects. However, the effect and safety of melatonin should be carefully monitored when melatonin is used clinically.

## Bacteriostatic Actions of Melatonin *In Vitro* and Their Underlying Mechanisms

Historically, a great diversity of studies aimed to investigate whether melatonin does possess bacteriostatic actions. Initially, melatonin has only been shown to strongly reduce the lipid levels of the yeast *Candida albicans* (*C. albicans*) (90). Subsequently, one comparative study found that some pineal indoles that share a similar structure with melatonin possess antibacterial activities. For instance, 5-methoxytryptamine (5-MT), which is both a metabolite and precursor of melatonin (65), exhibits antibacterial actions against *S. aureus* and *Bacillus subtilis* (*B. subtilis*), and 6-methoxy-2-benzoxazolinone (6-MBOA) against *Proteus vulgaris* (*P. vulgaris*). However, no corresponding antibacterial effect was observed in the case of melatonin (91). Later, it has been reported that melatonin (31.25~125 mg/mL: 0.13-0.53 mM) is able to suppress the growth of PGPB (e.g., *S. aureus*) and PGNB (e.g., *P. aeruginosa* and *A. baumannii*) by reducing key intracellular substrates *in vitro* (54). Intriguingly, melatonin could also inhibit the growth of the atypical GPB *M. tuberculosis*, while the exact mechanism has not yet been identified (92). In this section, we present further evidence for antibacterial activities of melatonin against PGPBs and PGNBs *in vitro*, and we also discuss the potential mechanisms.

The antimicrobial resistance among GPBs (chiefly *S. aureus*, *Enterococcus faecium*, *Enterococcus faecalis* and *Streptococcus pneumoniae*) has become a serious threat to public health, spurring the development of new compounds against infections (93, 94). In an early study, melatonin had been shown to moderately inhibit the growth of *S. aureus* (95). This was later also reported for the methicillin-resistant *S. aureus* (MRSA), but this required concentrations in the millimolar range (54). However, another investigation showed that the antibacterial action against *S. aureus* and/or MRSA of flouroquinolones was substantially diminished by melatonin *via* reduction of oxidative stress in the bacterial cells (96). Additional bacteria had been also tested in some studies (54, 95, 96), but melatonin’s efficiency against other important PGPBs, such as *Enterococcus faecium*, *Enterococcus faecalis* and *Streptococcus pneumoniae* remains to be demonstrated.

PGNBs are also a major challenge in public health, such as *P. aeruginosa* (97) and *A. baumannii* (98). *P. aeruginosa* is one of the main opportunistic pathogenic bacteria in hospitals (99, 100), which often results in post-operative (101) or post-burn wound infections (102), bacteremia (103) and sepsis (104). Antimicrobial resistance also exists in *P. aeruginosa*, it has been discovered that melatonin has a direct suppressive role in carbapenem-resistant *P. aeruginosa* (54). CBR-4830 (a tricyclic indole analog which shows chemical similarity to melatonin), was supposed to suppress the growth of *P. aeruginosa* (105). *A. baumannii*, is a conditional pathogen (106), which often leads to pneumonia (107), meningitis (108) and bacteremia (109). It causes hospital infection frequently (110) and is often resistant to a variety of antimicrobial agents (111), bringing great difficulties to the clinical treatment. Fortunately, melatonin effectively inhibits the growth of *A. baumannii* (54), suggesting it may be suitable as adjunctive therapy in hospital infection. Similarly, we also found that melatonin could inhibit the growth of enterotoxigenic *Escherichia coli* (ETEC) and *Pasteurella multocida* serotype A strain CQ2 (PmCQ2) (unpublished data). Of note, melatonin was reported to moderately reduce surface hydrophobicity, a characteristic associated with the colonization of mammalian epithelia, inhibiting the adherence of *E.coli*; however, the reduction of surface hydrophobicity in *E. coli* remained at borderline and was only observed at high concentrations (0.2 mM) (112). In contrast, another study by the same group reported that melatonin instead increased cell surface hydrophobicity of *Neisseria meningitidis* (113). Thus, the influence of melatonin on surface hydrophobicity is obviously variable, which would require a convincing explanation, especially as the authors had tried to relate these effects to melatonin’s antioxidant properties (112).

*M. tuberculosis*, an atypical PGPB whose cell wall is coated by mycolic acid (26), is the main pathogen of tuberculosis (TB) in humans (25, 114) and various mammals (115, 116). In addition to dormancy and persistence, drug resistance is a major obstacle in the treatment of TB (117). Therefore, it is urgent to seek for sensitive anti-TB drugs or methods to enhance the sensibility of existing antimicrobial agents. In the earlier literatures, the protective function of melatonin against *M. tuberculosis* was evaluated with isoniazid *in vitro*, experiments that did not reveal significant growth inhibitions by either isoniazid (0.005 to 0.01 μg/mL) or melatonin (0.26 nM to 2.6 nM) alone, but by a combination of isoniazid (0.005 μg/mL) and melatonin (0.01 mg/mL) (61, 92). Furthermore, the bacteriostatic capacity of melatonin was also reported for *M. tuberculosis* (3×10^7^ CFU/mL, 3×10^5^ CFU/mL, 3×10^3^ CFU/mL) treated with different concentrations of melatonin (0.05, 0.1, 0.2 and 0.4 mg/mL) *in vitro* (118). However, the extent of inhibition was not quantified in that study. Collectively, melatonin can affect pathogenic bacteria in some cases directly (e.g., inhibition of growth and adherence), which has been summarized in **Table 1**. It is likely concluded that the effectiveness of melatonin on pathogenic bacteria is obvioulsy variable, and may depend largely on its working concentrations. Current studies have centered on melatonin’s antibacterial activities on GNB, while the bacteriostatic capability of melatonin against other specific bacteria (especially antimicrobial resistant-GNB) is still poorly characterized.

**Table 1:** The antibacterial action of melatonin *in vitro* on different bacteria.

Bacteria	Dosage	Time	Effects	Ref.
Carbapenem-resistant *P. aeruginosa*	31.25 or 125 μg/mL (0.13 or 0.53 mM)	24 or 48 h	Inhibition of carbapenem-resistant *P. aeruginosa* growth	(54)
Carbapenem-resistant *A. baumannii*	31.25 or125 μg/mL (0.13 or 0.53 mM)	24 or 48h	Inhibition of crbapenem-resistant *A. baumannii* growth	(54)
Methicillin-resistant *S. aureus*	125 or 250 μg/mL (0.53 or 1.07 mM)	24 or 48h	Inhibition of methicillin-resistant *S. aureus* growth	(54)
*S. aureus* ATCC 29123	125 or 250 μg/mL (0.53 or 1.07 mM)	24 or 48h	Inhibition of *S. aureus* (ATCC 29123) growth	(54)
*P. aeruginosa* ATCC 27853	125 or 250 μg/mL (0.53 or 1.07 mM)	24 or 48h	Inhibition of *P.aeruginosa* (ATCC 27853) growth	(54)
*M. tuberculosis* H37Rv	26.0 nM 0.01 to 10 mM	unknown	Inhibition of *M. tuberculosis* H37Rv growth The antibacterial efficacy of melatonin with isoniazid increased at least a threefold	(92)
*M. tuberculosis bovis* BCG	0.13 to 10 mM	unknown	Inhibitin of *M. bovis* BCG growth	(92)
*Multidrug-resistant M. tuberculosis* (TBRI 40 and TBRI 204)	0.01 to 10 mM	unknown	Inhibition of multidrug-resistant *M. tuberculosis* (TBRI 40 and TBRI 204) growth	(92)
*Xanthomonas oryzae pv. Oryzae* (Xoo)	200 μg/mL	12, 21 or 24 h	Inhibition of Xoo proliferation, motility and biofilm formation Alteration of Xoo cells length Downregulation of mRNA expression of genes involved in cell division, carbohydrate metabolism and amino acid metabolism	(119)
*Xanthomonas oryzae pv. Oryzicola* Xoc)	200 μg/mL	24 h	Inhibition of Xoc growth, the motility and biofilm formation Reduction of mRNA expression of genes related to toxin and cell division	(120)

P. aeruginosa, Pseudomonas aeruginosa; A. baumannii, Acinetobacter baumannii; S. aureus, Staphylococcus aureus; M. tuberculosis, Mycobacterium tuberculosis; B. anthracis, Bacillus anthracis.

**Figure 1:**
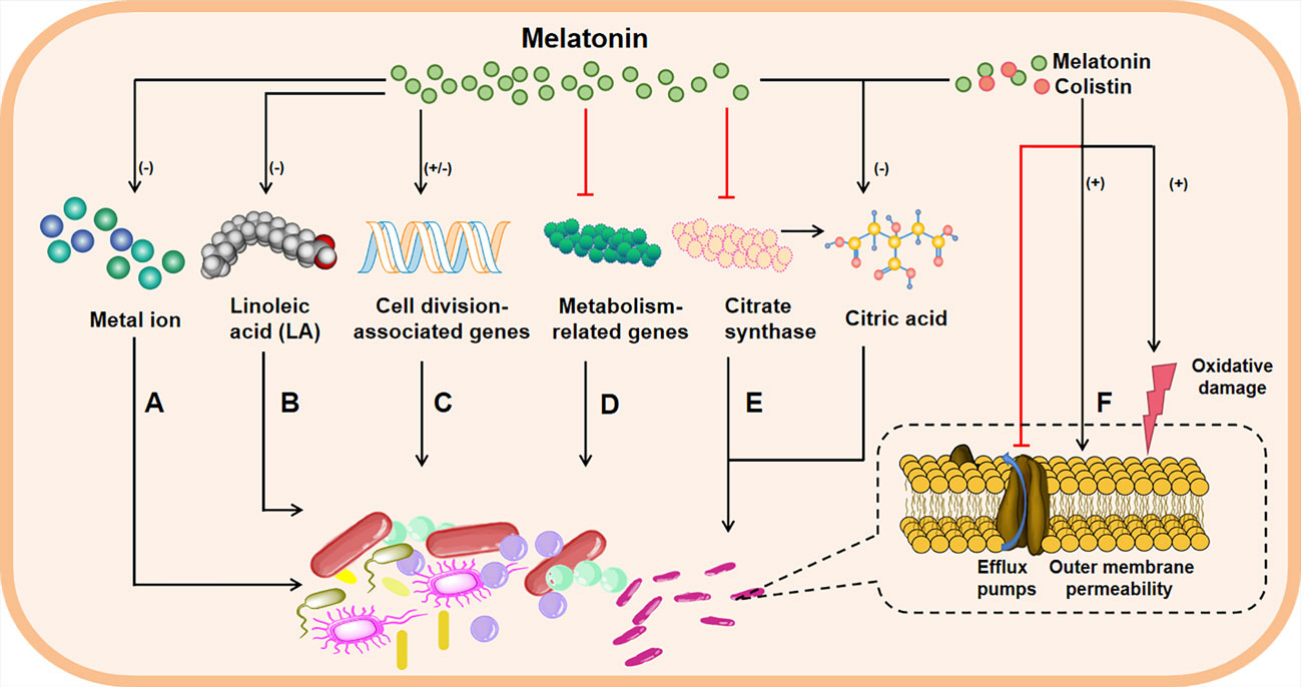
The possible bacteriostatic mechanisms of melatonin *in vitro.* Melatonin inhibits the growth of bacteria through these avenues: reducing cytoplasmic availability of metal ion **(A)**; interfering cell wall formation, for example, melatonin restricts the absorb of Linoleic acid (LA), which makes up the cell wall, thus limiting cell proliferation **(B)**; melatonin can also regulate the expressions of genes associated with cell division **(C)** or suppress the content and activity of metabolism-related enzymes **(D)** to inhibit bacterial growth and proliferation; melatonin resists against Gram-negative bacteria through inhibiting bacterial citrate synthase and reducing the synthesis of citric acid **(E)**, besides, when melatonin is combination with colistin, bacterial outer membrane permeability and oxidative damage are enhanced, and the effect of efflux pumps is inhibited **(F)**, leading to increased bacteria damage. Note: minus in parentheses represents decrease; plus in parentheses represents increase; and the red line represents inhibition; the black arrow indicates activation.

As mentioned above, melatonin could exhibit bacteriostatic action against pathogenic bacteria; however, the potential mechanisms are not fully understood. Actually, there are two main hypotheses about bacteriostatic mechanisms of melatonin *in vitro*. It has been well documented that the growth of bacteria urgently requires metals, particularly free iron (121). Considering that melatonin has a high metal binding capacity, including iron, copper and zinc (122), thus, the addition of melatonin may reduce cytoplasmic availability of metal ions in bacteria to achieve the bacteriostatic effects (**Figure 1A**). Moreover, the membrane of bacteria is imbued with phospholipids (123, 124), and study has shown that melatonin can restrict the absorb of Linoleic acid (LA) used for facilitating cell proliferation (125). Therefore, melatonin may inhibit proliferation of bacteria by blocking the getting of bacterial growth factors (**Figure 1B**). These convincing findings indicate that melatonin inhibits the bacterial growth may through reducing intracellular substrates (54). Interestingly, a recent study declared that melatonin inhibits bacterial growth and proliferation by regulating the expression of genes associated with cell division (**Figure 1C**) and suppressing contents as well as activities of metabolism-related enzymes (**Figure 1D**) (119). These interesting results imply that melatonin could affect bacteria physiological condition by intrinsic molecular mechanisms; however, the experimental validation is still needed. Of note, our recent findings also demonstrated that melatonin exerts antibacterial activity against GNB (e.g., *Klebsiella*, *Pasteurella multocida* and *Pseudomonas aeruginosa*) through specifically inhibiting the activity of bacterial citrate synthase (**Figure 1E**), and the combination of colistin with melatonin enhances bacterial outer membrane permeability, oxidative damage and inhibits the effect of efflux pumps (**Figure 1F**) (126).

## Bacteriostatic Actions of Melatonin *In Vivo* and Their Underlying Mechanisms

Melatonin not only has antibacterial effect *in vitro*, but also plays a significant role in the clinical prevention and treatment of bacterial infections *in vivo* (61). The inhibitory effects of melatonin *in vivo* includes both on PGPB [e.g., *S. aureus* (56) and *S. pneumoniae* (127)] and PGNB [e.g., *E.coli* (128), *H. Pylori* (129) and *K. pneumonia* (130)]. For example, in many GNB-infected animal models (e.g., sepsis), melatonin has a favorable effect in improving survival rates, ameliorating tissue damage and reducing levels of pro-inflammation mediators (e.g., TNF-α and IFN-γ), and increasing levels of anti-inflammation mediators (e.g., IL-10) (131, 132). Notably, various signaling have been described to shape bacterial infection *in vivo* by melatonin, including NF-κB, Nrf2, NLRP3 and ROS pathways. In this section, firstly, we present the evidence from studies that have used melatonin to resist bacterial infections *in vivo*. Subsequently, we illustrate the possible mechanisms.

*S. aureus* always causes many serious infections in human (133, 134). Virtually, melatonin not only inhibits the growth of *S. aureus in vitro*, but also has significant defensive effects against *S. aureus*-induced infection *in vivo*. For example, melatonin reduces lipid peroxidation (LPO), catalase (CAT), neutrophil recruitment, and TNF-α, IFN-γ, IL-6, iNOS, COX-2 and C-reactive protein (CRP), while increases superoxide dismutase (SOD) and glutathione (GSH) in animals infected by *S. aureus* (5 × 10^6^ CFU/mL) (56). The pathogenicity of *S. pneumoniae* is second only to *S. aureus* in pyogenic cocci (135), which triggers major lobe pneumonia (136), meningitis (137), bronchitis (138). Fortunately, it has found that melatonin has a certain inhibitory effect on the infection of *S. pneumonia* (95). These aforementioned findings indicate that melatonin can target GPB to alleviate infections *in vivo*. However, it has been shown that melatonin therapy fails to reduce neuronal injury in *S. pneumoniae*-infected rabbit model, the possible reason may due to the 12 h delay in the administration of melatonin after the infection (139).

Historically, *E. coli* has been regarded as an integral part of the normal intestinal flora (140), and was considered to be non-pathogenic bacteria (141). Nevertheless, as the research moves along, some special serotypes of *E. coli* [e.g., ETEC (142), enterohemorrhagic *E. coli* (EHEC) (143) and enteropathogenic *E. coli* (EPEC) (144)] are shown to be pathogenic to humans and animals (especially infants and young animals) (145), causing severe diarrhea (146) and sepsis (147). Some studies have found that melatonin has a bacteriostatic effect in the animals infected by *E. coli*, for instance, melatonin reduces LPO, CAT, neutrophil recruitment, and TNF-α, IFN-γ, IL-6, iNOS, COX-2 and CRP, while increases SOD and GSH in animals infected by *E. coli* (2.5 × 10^7^ CFU/mL) (56, 148). *H. Pylori* is the only microbial species known to survive in the human stomach (149) to induce gastritis (150), gastrointestinal hemorrhage (151) and gastric lymphoma (152). It has been revealed that *H. Pylori* infection inhibits gastric mucosal melatonin synthesizing enzymes [e.g., arylalkylamine-N-acetyltransferase (AANAT) and N-acetylserotonin O-methyltransferase (ASMT)] expression (153). Interestingly, melatonin facilitates *H. pylori* eradication in patients with gastroduodenal ulcer by omeprazole treatment (154–156), indicating melatonin can be used as an adjuvant clinical drug against *H. Pylori* infection. Furthermore, *K. pneumonia* is also a member of PGNB, isolated from the sputum of patients with pneumonia (157), mainly leads to pneumonia (158), septicemia (159) or bacteremia (160), meningitis (161) and peritonitis (162). An experimental study showed that the supply of 100 mg/kg of melatonin can reduce pro-inflammatory cytokines, inhibit microglial activation, and counteract neurocognitive damage in *K. pneumoniae*-infected rats (130). Likewise, we also found that melatonin can inhibit macrophage-mediated excessive inflammatory responses in *Pasteurella multocida* (PmCQ2)-infected mice (unpublished data). Of note, like GPB *S. pneumoniae*, the effect of melatonin on inactivated *Pasteurella multocida* (P52 strain) vaccine-mediated immune responses is time-dependent evidenced by exogenous melatonin administration at 4 h post vaccination augments immune responses in rats in comparison to 16 h post vaccination (163, 164).

Notably, melatonin has been demonstrated to increase survival rates and improve organ function in several sepsis models (165, 166). The oxidative imbalance is one of the characteristics of sepsis (167), and mitochondria play key roles in regulating sepsis-related redox dysregulation (168). Mechanistically, melatonin can alleviate sepsis symptoms by preventing mitochondria dysfunction *via* ROS/RNS scavenging (169) and many other pathways (e.g., intra-mitochondrial SIRT3 and MAPK/ERK pathway) (170–176). Inflammation is essential for the host to resist infection by pathogenic bacteria, however, excessive inflammation is another characteristic of the initial stage of sepsis, leading to organ dysfunction and eventually death (177, 178).

Melatonin as a signal molecule of stress can be induced by the pathogenic (but not the beneficial) bacteria invasion and the increased melatonin level in hosts can improve the protective effects or tolerance to the bacteria (179). In addition to inhibiting pathogenic bacteria, melatonin has a beneficial effect on intestinal flora (180, 181). For example, melatonin reprogramming of gut microbiota improves lipid dysmetabolism to prevent obesity in mice (182–185). Moreover, melatonin contributes to reshape gut microbiota to alleviate neuroinflammation and metabolic disorder in DSS-induced depression rats (186). Furthermore, melatonin ameliorates ochratoxin A-induced liver inflammation, oxidative stress and mitophagy involving in intestinal microbiota in mice (187). Altogether, as summarize in **Table 2**, melatonin may function as a novel compound to resist pathogenic bacterial infections *in vivo*. However, the defensive effects of melatonin against pathogenic bacteria are likely dose- and/or time-dependent.

**Table 2:** The antibacterial action of melatonin *in vivo* infected with different bacteria.

Bacteria	Dosage	Time	Effects	Ref.
*S. aureus*	10 mg/kg	At 17:00, 17:30, 18:00	Reducing expression of LPO, CAT, iNOS, COX-2 and production of TNF-α, IFN-γ, IL-6, CRP, increasing the production SOD and GSH	(56)
*S. pneumoniae*	2 mg/mL	40 h	Suppressing bacterial growth at a high concentration	(95)
*E. coli*	10 mg/kg or 1nM	At 17:00, 17:30 and 18:00 or 70 s	Reducing expression of LPO, CAT, iNOS, COX-2 and production of TNF-α, IFN-γ, IL-6, CRP, increasing the production SOD and GSH	(56, 148)
*H. Pylori*	5 mg or 3 mg	21 days	Increasing efficacy of *H. pylori* elimination, Accelerating duodenal ulcer recovery	(154–156)
*K. pneumonia*	100 mg/kg	24 h	Counteracting neurocognitive damage inhibiting microglial activation, and reducing pro-inflammatory cytokine levels	(130)
*Pasteurella multocida* (P52)	100mg/kg	4 h	Exogenous melatonin at 4 h post vaccination augments immune responses in rats.	(163)
*Pasteurella multocida* (PmCQ2)	30 mg/kg, 60 mg/kg, 120mg/kg	12 h, 16 h, 24 h, 32 h	Inhibiting macrophage-mediated excessive inflammatory responses	Unpublished

S. aureus, Staphylococcus aureus; S. pneumonia, Streptococcus pneumonia; E. coli, Escherichia coli; H. Pylori, Helicobacter Pylori; K. pneumonia, Klebsiella pneumonia; LPO, lipid peroxidation; CAT, catalase; iNOS, inducible nitric oxide synthase, CRP, C-reactive protein; SOD, superoxide dismutase; GSH, glutathione.

**Figure 2:**
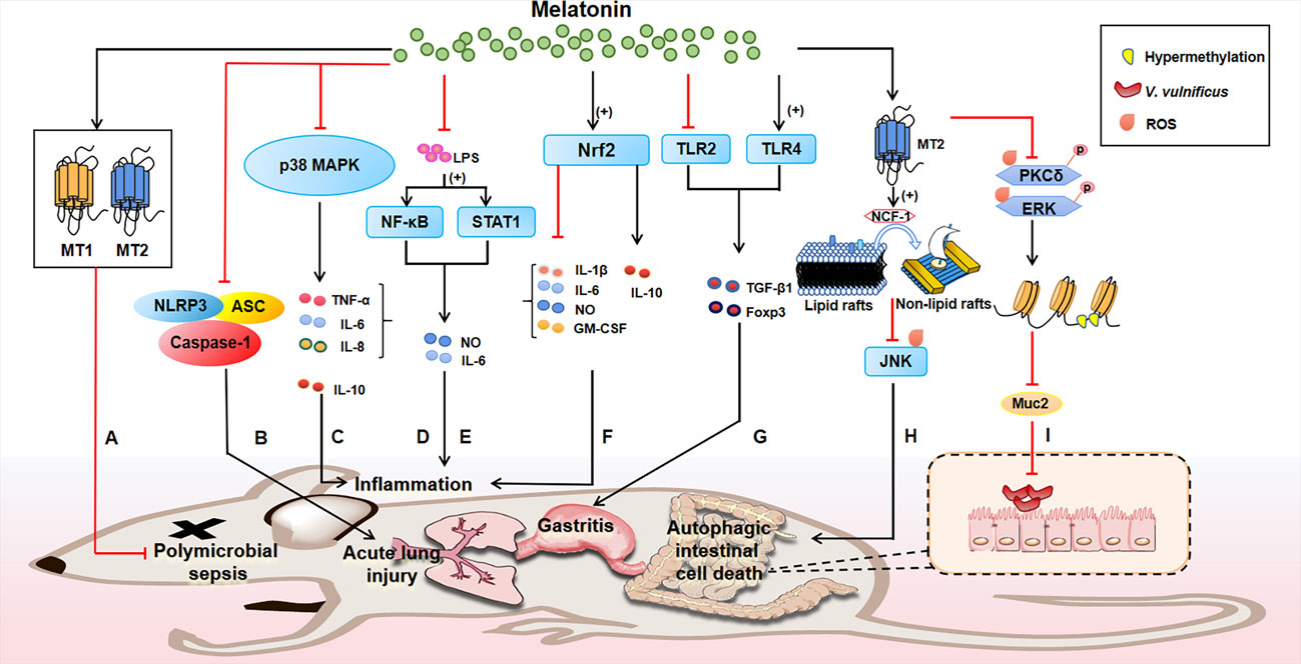
The bacteriostatic mechanisms of melatonin in animals. Melatonin receptors (e.g., MT1 and MT2) play a great role in mediating the functions of melatonin. Melatonin reduces polymicrobial sepsis through MT1 and MT2, thus improving the survival rate of mice **(A)**; notably, the mechanisms whereby melatonin exerts bacteriostatic action *in vivo* are tightly associated with the immune responses, for instance, melatonin inhibits NLRP3 inflammasome to alleviate acute lung injury **(B)**; melatonin reduces pro-inflammatory cytokines, increases anti-inflammatory cytokines and improves survival through p38MAPK signaling pathway **(C)**; melatonin blocks LPS-induced activation of NF-κB **(D)** and STAT1 **(E)**, thus inhibiting the production of inflammatory factors to modulate inflammation. Likewise, suppression of inflammation also occurs when melatonin activates Nrf2, evidenced by reduced pro-inflammatory mediators (e.g., IL-1β, IL-6, NO and GM-CSF) and increased anti-inflammatory cytokine (e.g., IL-10) **(F)**; melatonin treatment can target TLR to alleviate *H*. *pylori*-induced gastritis by promoting TLR4 and inhibiting TLR2 to regulate TGF-β1 and Foxp3 expression **(G)**; of note, melatonin could be a therapeutic alternative agent to fight bacterial infections due to its antioxidant function. For example, melatonin signaling *via* MT2 promotes NCF-1 recruitment from lipid rafts to non-lipid rafts to block the ROS-mediated JNK pathway, preventing autophagic intestinal cell death **(H)**; moreover, MT2 signaling inhibits the ROS-mediated phosphorylation of PKCδ and ERK to reduce region-specific hypermethylation in the Muc2 promoter, combating *V. vulnificus* infection **(I)**. Note: plus in parentheses represents increase; and the red line represents inhibition; the black arrow indicates activation.

Notably, the mechanisms whereby melatonin exerts bacteriostatic action *in vivo* are tightly associated with the immune responses, for example, during polymicrobial infection, melatonin treatment could promote the development of the neutrophil extracellular trap (NET), whereas inhibits the phagocytic activities of neutrophils (188). Indeed, mounting evidences suggest that melatonin always exerts its physiological effects *via* its receptors (e.g., MT1 and MT2). It has been discovered that melatonin is able to improve the survival rate of polymicrobial sepsis of mice through MT1 and MT2 receptor (**Figure 2A**) (189). Melatonin also alleviates acute lung injury induced by LPS *via* inhibiting the activation of NLRP3 inflammasome (**Figure 2B**) (190). Melatonin could dose-dependently reduce pro-inflammatory cytokine TNF-α, IL-6 and IL-8, increase anti-inflammatory cytokine IL-10 and improve survival, which are associated with p38MAPK and NF-κB signaling pathway (173, 191–195) (**Figure 2C**). Moreover, melatonin could block LPS (which is from *P. intermedia*)-induced activation of NF-κB signaling (**Figure 2D**) and STAT1 pathway (**Figure 2E**), thereby inhibiting the production of inflammatory mediators (e.g., NO and IL-6) (196, 197). Likewise, it has found that melatonin can reduce pro-inflammatory mediators [e.g., IL-1β, IL-6, NO and granulocyte-monocyte colony-stimulating factor (GM-CSF)], while increase anti-inflammatory cytokine (e.g., IL-10) by activating Nrf2 pathway (**Figure 2F**) (198). Actually, melatonin treatment can also alleviate *H. pylori*-induced gastritis through regulating TGF-β1 and Foxp3 expression *via* the suppression of TLR2 and activation of TLR4 (**Figure 2G**) (129), although the molecular target of melatonin in the TLR signaling warrants further investigation.

Moreover, it should be noted that melatonin could be a therapeutic alternative agent to fight bacterial infections due to its antioxidant function. Intriguingly, melatonin inhibits apoptotic cell dealth in colonic epithelial cells induced by *Vibrio vulnificus* VvhA *via* MT2 (199). Mechanistically, melatonin signaling *via* MT2 stimulates NCF-1 recruitment into non-lipid rafts from lipid rafts to block the ROS-mediated JNK pathway, preventing rVvhA-induced apoptosis and autophagic intestinal cell death (**Figure 2H**). Similarly, melatonin treatment maintains the expression level of Muc2 in the intestine of *V. vulnificus*-infected mouse. Mechanistically, melatonin inhibits the ROS-mediated phosphorylation of PKCδ and ERK responsible for region-specific hypermethylation in the Muc2 promoter *via* MT2 receptor, restoring the level of Muc2 production in intestinal epithelial cells to resist *V. vulnificus* infection (**Figure 2I**) (200).

Collectively, melatonin could exert its bacteriostatic action *in vivo* by various potential mechanisms, including NF-κB, STAT1, Nrf2, TLR2/4, and ROS signaling (**Figure 2**). These aforementioned findings may provide promising strategies for controlling many diseases of public health importance. It should not be neglected that melatonin has a significant antibacterial effect *in vitro*; thus, whether the powerful effect of melatonin *in vivo* is also directly inhibiting bacterial growth remains further exploration.

## Bacteriostatic Actions of Melatonin in Plants and Their Underlying Mechanisms

**Figure 3:**
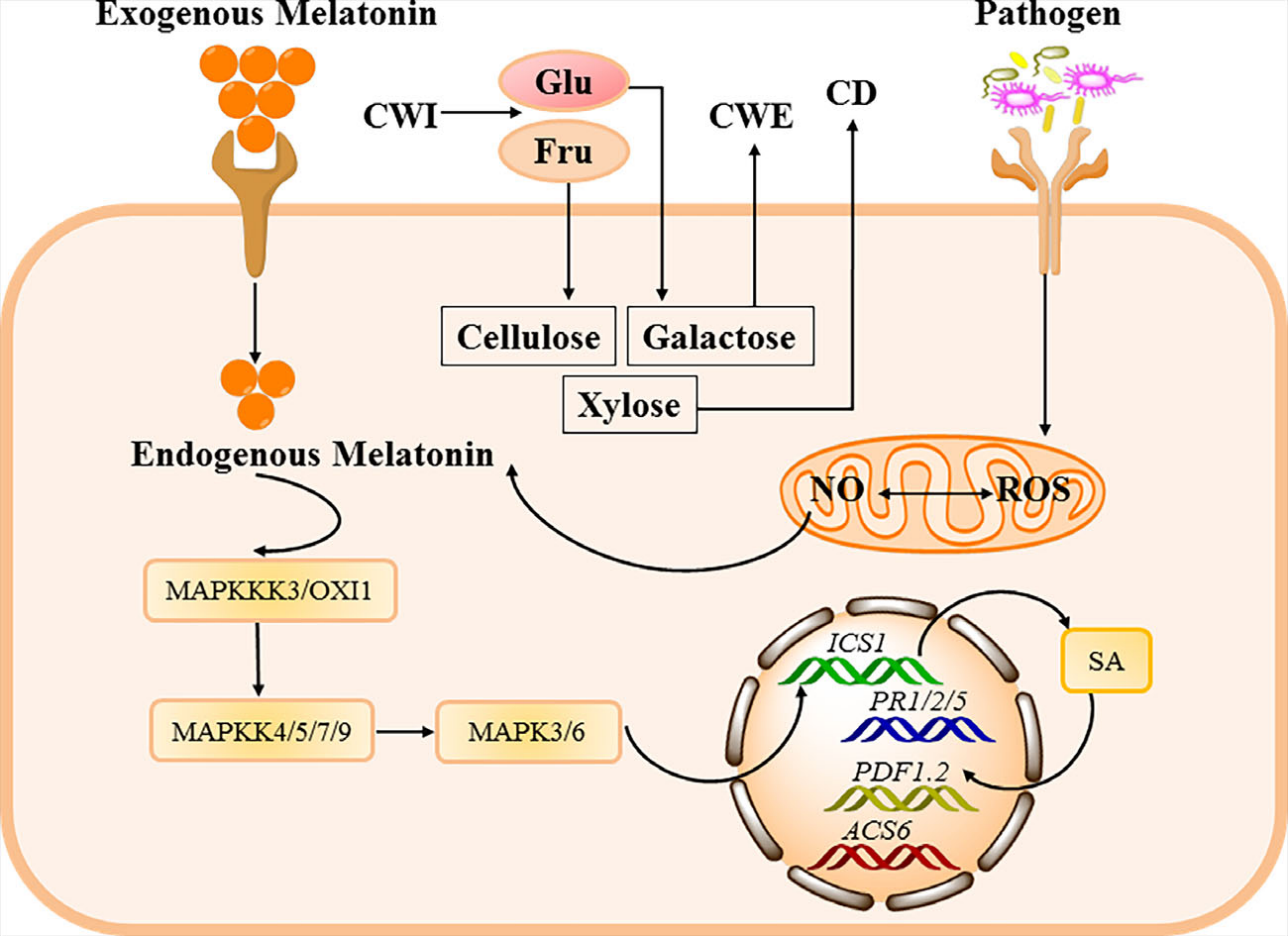
The bacteriostatic mechanisms of melatonin in plants. Antibacterial mechanisms against plant pathogenic bacteria are characterized by up-regulation of defense genes such as *plant defensin1.2* (*PDF1.2*), *plant resistance 1/2/5* (*PR1/2/5*), and *1-aminocyclopropane-1-carboxylate synthase 6* (*ACS6*) through several signal transduction pathways, including augmentation of NO levels in plants, which collaborates with melatonin in up-regulating SA. Moreover, melatonin can stimulate mitogen-activated protein kinases (MAPKs) cascades, which in turn up-regulate SA biosynthesis gene *isochorismate synthase 1* (*ICS1*). Additionally, high cell wall invertase (CWI) activity within melatonin-treated *Arabidopsis* leads to improved cell wall strengthening and callose-depositing factors (cellulose, xylose, and galactose).

In addition to the significant bacteriostatic functions of melatonin *in vitro* and in animals, melatonin also exerts similar function against pathogenic bacterial infections in plants (201, 202). It has found that melatonin treatment inhibits the growth, motility and capsule formation of the bacterium *Xanthomonas oryzae pv. oryzae* (Xoo) (119), which leads to bacterial blight in rice (203, 204). Melatonin increases the resistance to *Verticillium dahlia* in cotton by regulating lignin and gossypol biosynthesis (205), although the molecular mechanisms are still not available. Authentically, it has been demonstrated that Serotonin N-acetyltransferase (SNAT)-deficient *Arabidopsis* shows lower melatonin levels and exhibits susceptibility to pathogen infection (206). Conformably, melatonin can trigger defense responses against *Pst* DC3000 infection in *Arabidopsis* and/or tobacco (55, 207, 208). Mechanistically, melatonin-induced rise of NO that favors in the expression of salicylic acid (SA)-related genes (e.g., *AtEDS1*, *AtPAD4*, *AtPR1*, *AtPR2* and *AtPR5*), conferring improved disease resistance against *Pst* DC3000 infection in *Arabidopsis* (**Figure 3**); however, the beneficial effects of melatonin could be jeopardized by using a NO scavenger (cPTIO) and/or lost in NO-deficient mutants of *Arabidopsis* (209). Furthermore, it has been discovered that melatonin can active MPK3 and MPK6 signaling (members of MAPKs), which are independent of G-protein and Ca^2+^ signaling, and the inhibition of MPK3 and/or MPK6 induces reduced expression of defense and pathogen resistance-related genes (e.g. *PR1*, *PR2*, and *PR5*) (55). Indeed, melatonin activates MPK3 and MPK6 *via* four MKKs (i.e., MKK4/5/7/9) (210), and MAPKKK 3 and OXI 1 (oxidative signal-inducible 1) kinases are responsible for triggering melatonin-induced defense signaling pathways (211). Therefore, melatonin could mediate pathogen resistance in *Arabidopsis* and tobacco by activating MAPKs signaling *via* MKK4/5/7/9-MPK3/6 cascades through the activation of MAPKKK 3 and OXI 1 (**Figure 3**). In conclusion, melatonin plays a critical bacteriostatic role in *Pst* DC3000-infected *Arabidopsis* by MAPKs pathway. Moreover, melatonin could improve cell wall strengthening and callose-depositing factors (cellulose, xylose, and galactose) by increasing cell wall invertase (CWI) activity in *Arabidopsis* infected with *Pst. DC3000* (**Figure 3**) (207). However, whether melatonin could function as a signaling molecule in modulating defense responses of other plants infected by various pathogenic bacteria are still poorly defined.

## Concluding Remarks

Melatonin has multifarious functions, like circadian rhythm regulation (212, 213); anti-inflammatory/anti-tumor effects (214, 215); and, with particular relevance to this article, anti-bacterial function. Here, we summarize melatonin can directly influence bacteria *in vitro* (e.g., inhibition of growth) by reducing intracellular substrates. Considering that intestinal bacteria, *Enterobacter aerogenes*, responds to the melatonin by an increase in swarming activity, which is expressed rhythmically (216); thus, it is meaningful to further investigate the potential effects of melatonin in synchronizing bacterial rhythms *in vitro*. Notably, melatonin resists pathogenic bacterial infections *in vivo* by various pathways, such as NF-κB, TLR2/4, and ROS. Other pathways seem to get involved as well, but further experimental validation is needed. Furthermore, intestinal microbiota play a crucial role in the progress of different diseases, including bacterial infections *in vivo* (183, 217); therefore, it is necessary to further investigate the intestinal microbiota-mediated defensive roles of melatonin in attenuating bacterial infections *in vivo*. Melatonin serves as a signaling molecule to resist bacterial infections in *Arabidopsis* mainly through MAPK pathway, whether other specific pathways connecting to the bacteriostatic actions of melatonin in other plants or even other specific bacteria also remain an open question.

In addition to its significant antibacterial effect, it is thought that melatonin plays crucial roles in the fighting viral infections (61). Melatonin was found to effective against Ebola hemorrhagic shock syndrome by inhibiting Rho/ROCK signaling (218). Melatonin was also shown to protect mice infected with the Venezuelan equine encephalitis virus (VEEV) by reducing viral loads, brain apoptosis and oxidative stress (219, 220). Additionally, melatonin also was reported to exert protective and therapeutic effects against hemorrhagic disease virus (221), respiratory syncytial virus (RSV) (222), aleutian disease virus (223) and influenza virus (224). Recently, melatonin was proposed to be a potential candidate drug as an adjuvant treatment for patients with COVID-19 based on its antioxidant, anti-inflammatory and immunomodulatory properties (81, 225–227).

Overall, this review highlights melatonin as a novel and feasible preventive and therapy to tackle the increasing threat by bacterial infections. Based on the various physiological/pharmacological functions of melatonin and its significant anti-pathogenic effects, melatonin can be possibly used as clinical agent against pathogens and even viruses in the future.

## Author Contributions

FH, XW, and YX designed and wrote the review article. PL, SC, RH, and YX revised the review article. FH, XW, QZ, YL, YY, PL, SC, and YP helped with designing figures and finding relevant literatures. YX approved the final manuscript. All authors contributed to the article and approved the submitted version.

## Funding

This work was supported by Ministry of China Agriculture & Rural Affairs and Finance: the National Modern Agricultural Industry Technology System (Beef/Yak Cattle, CARS-37), and Chongqing Science & Technology Commission (cstc2017jcyjAX0288).

## Conflict of Interest

The authors declare that the research was conducted in the absence of any commercial or financial relationships that could be construed as a potential conflict of interest.

## Abbreviations

GPB, gram positive bacteria; GNB, gram negative bacteria; PGPB, pathogenic gram positive bacteria; PGNB, Pathogenic gram negative bacteria; *S. aureus*, *Staphylococcus aureus*; *S. pneumoniae*, *Streptococcus pneumoniae*; *B. anthracis*, *Bacillus anthracis*; *E. coli*, *Escherichia coli*; *H. pylori*, *Helicobacter pylori*; *P. aeruginosa*, *Pseudomonas aeruginosa*; *A. baumannii*, *Acinetobacter baumannii*; *K. pneumoniae*, *Klebsiella pneumoniae*; *M. tuberculosis*, *Mycobacterium tuberculosis*; *C. albicans*, *Candida albicans*; *B. subtilis*, *Bacillus subtilis*; *P. vulgaris*, *Proteus vulgaris*; MRSA, methicillin-resistant *S. aureus*; ALS, Amyotrophic lateral sclerosis; 5-MT, 5-methoxytryptamine; 6-MBOA, 6-methoxy-2-benzoxazolinone; ETEC, enterotoxigenic *Escherichia coli*; EHEC, enterohemorrhagic *E. coli*; EPEC, enteropathogenic *E. coli*; Xoo: *Xanthomonas oryzae pv. Oryzae*; AANAT, arylalkylamine-N-acetyltransferase; ASMT, N-acetylserotonin O-methyltransferase; PmCQ2, *Pasteurella multocida* serotype A strain CQ2; TB, tuberculosis; LA, Linoleic acid; LPO, peroxidation; CAT, catalase; CRP, C-reactive protein; SOD, superoxide dismutase; GSH, glutathione; NET, neutrophil extracellular trap; GM-CSF, granulocyte-monocyte colony-stimulating factor; OXI 1, oxidative signal-inducible.

## References

[1] Trdá L Boutrot F Claverie J Brulé D Dorey S Poinssot B. Perception of Pathogenic or Beneficial Bacteria and Their Evasion of Host Immunity: Pattern Recognition Receptors in the Frontline. Front Plant Sci (2015) 6:219. 10.3389/fpls.2015.00219 25904927 PMC4389352

[2] Jawad KI Razavi SH. Characterization of Lactobacillus Plantarum as a Potential Probiotic In Vitro and Use of a Dairy Product (Yogurt) as Food Carrier. Appl Food Biotechnol (2017) 4:11–8. 10.22037/afb.v4i1.13738

[3] Kok CR Hutkins R. Yogurt and Other Fermented Foods as Sources of Health-Promoting Bacteria. Nutr Rev (2018) 76:4–15. 10.1093/nutrit/nuy056 30452699

[4] Broadbent JR Barnes M Brennand C Strickland M Houck K Johnson ME. Contribution of Lactococcus Lactis Cell Envelope Proteinase Specificity to Peptide Accumulation and Bitterness in Reduced-Fat Cheddar Cheese. Appl Environ Microbiol (2002) 68:1778–85. 10.1128/aem.68.4.1778-1785.2002 PMC123837 11916696

[5] Abdelfatah EN Mahboub HHH. Studies on the Effect of Lactococcus Garvieae of Dairy Origin on Both Cheese and Nile Tilapia (O. Niloticus). Int J Vet Sci Med (2018) 6:201–7. 10.1016/j.ijvsm.2018.11.002 PMC6286421 30564596

[6] Mustapha NA Hu A Yu CP Sharuddin SS Ramli N Shirai Y. Seeking Key Microorganisms for Enhancing Methane Production in Anaerobic Digestion of Waste Sewage Sludge. Appl Microbiol Biotechnol (2018) 102:5323–34. 10.1007/s00253-018-9003-8 29696331

[7] Bampidis V Azimonti G Bastos ML Christensen H Dusemund B Kos Durjava M. Safety and Efficacy of Monosodium L-Glutamate Monohydrate Produced by Corynebacterium Glutamicum KCCM 80188 as a Feed Additive for All Animal Species. Efsa J (2020) 18:e06085. 10.2903/j.efsa.2020.6085 32874293 PMC7448065

[8] Baek JH Kim KH Moon JY Yeo SH Jeon CO. Acetobacter Oryzoeni Sp. Nov., Isolated From Korean Rice Wine Vinegar. Int J Syst Evol Microbiol (2020) 70:2026–33. 10.1099/ijsem.0.004008 31995463

[9] Gueniche A Benyacoub J Philippe D Bastien P Kusy N Breton L. Lactobacillus Paracasei CNCM I-2116 (ST11) Inhibits Substance P-Induced Skin Inflammation and Accelerates Skin Barrier Function Recovery In Vitro. Eur J Dermatol (2010) 20:731–7. 10.1684/ejd.2010.1108 20965806

[10] Barko PC McMichael MA Swanson KS Williams DA. The Gastrointestinal Microbiome: A Review. J Vet Intern Med (2018) 32:9–25. 10.1111/jvim.14875 29171095 PMC5787212

[11] Huang M Cai S Su J. The Pathogenesis of Sepsis and Potential Therapeutic Targets. Int J Mol Sci (2019) 20:5376. 10.3390/ijms20215376 PMC6862039 31671729

[12] Marangu D Zar HJ. Childhood Pneumonia in Low-and-Middle-Income Countries: An Update. Paediatr Respir Rev (2019) 32:3–9. 10.1016/j.prrv.2019.06.001 31422032 PMC6990397

[13] Koelman DLH Brouwer MC van de Beek D. Targeting the Complement System in Bacterial Meningitis. Brain (2019) 142:3325–37. 10.1093/brain/awz222 PMC6821383 31373605

[14] Tripathi N Sapra A. Gram Staining. In: Statpearls. Treasure Island (FL: StatPearls Publishing (2021). Copyright © 2021, StatPearls Publishing LLC.

[15] Wang W Wang Y Lin L Song Y Yang CJ. A Tridecaptin-Based Fluorescent Probe for Differential Staining of Gram-Negative Bacteria. Anal Bioanal Chem (2019) 411:4017–23. 10.1007/s00216-018-1465-0 30415405

[16] Becerra SC Roy DC Sanchez CJ Christy RJ Burmeister DM. An Optimized Staining Technique for the Detection of Gram Positive and Gram Negative Bacteria Within Tissue. BMC Res Notes (2016) 9:216. 10.1186/s13104-016-1902-0 27071769 PMC4828829

[17] Jenul C Horswill AR. Regulation of Staphylococcus Aureus Virulence. Microbiol Spectr (2019) 7:1–21. 10.1128/microbiolspec.GPP3-0031-2018 PMC6452892 30953424

[18] Kadioglu A Weiser JN Paton JC Andrew PW. The Role of Streptococcus Pneumoniae Virulence Factors in Host Respiratory Colonization and Disease. Nat Rev Microbiol (2008) 6:288–301. 10.1038/nrmicro1871 18340341

[19] Norris MH Kirpich A Bluhm AP Zincke D Hadfield T Ponciano JM. Convergent Evolution of Diverse Bacillus Anthracis Outbreak Strains Toward Altered Surface Oligosaccharides That Modulate Anthrax Pathogenesis. PLoS Biol (2020) 18:e3001052. 10.1371/journal.pbio.3001052 33370274 PMC7793302

[20] Rueter C Bielaszewska M. Secretion and Delivery of Intestinal Pathogenic Escherichia Coli Virulence Factors Via Outer Membrane Vesicles. Front Cell Infect Microbiol (2020) 10:91. 10.3389/fcimb.2020.00091 32211344 PMC7068151

[21] Ji J Yang H. Using Probiotics as Supplementation for Helicobacter Pylori Antibiotic Therapy. Int J Mol Sci (2020) 21:1136. 10.3390/ijms21031136 PMC7037652 32046317

[22] Azam MW Khan AU. Updates on the Pathogenicity Status of Pseudomonas Aeruginosa. Drug Discov Today (2019) 24:350–9. 10.1016/j.drudis.2018.07.003 30036575

[23] Harding CM Hennon SW Feldman MF. Uncovering the Mechanisms of Acinetobacter Baumannii Virulence. Nat Rev Microbiol (2018) 16:91–102. 10.1038/nrmicro.2017.148 29249812 PMC6571207

[24] Martin RM Bachman MA. Colonization, Infection, and the Accessory Genome of Klebsiella Pneumoniae. Front Cell Infect Microbiol (2018) 8:4. 10.3389/fcimb.2018.00004 29404282 PMC5786545

[25] Le Chevalier F Cascioferro A Majlessi L Herrmann JL Brosch R. Mycobacterium Tuberculosis Evolutionary Pathogenesis and its Putative Impact on Drug Development. Future Microbiol (2014) 9:969–85. 10.2217/fmb.14.70 25302954

[26] Fu LM Fu-Liu CS. Is Mycobacterium Tuberculosis a Closer Relative to Gram-Positive or Gram-Negative Bacterial Pathogens? Tuberculosis (Edinb) (2002) 82:85–90. 10.1054/tube.2002.0328 12356459

[27] Jones KE Patel NG Levy MA Storeygard A Balk D Gittleman JL. Global Trends in Emerging Infectious Diseases. Nature (2008) 451:990–3. 10.1038/nature06536 PMC5960580 18288193

[28] Morens DM Folkers GK Fauci AS. The Challenge of Emerging and Re-Emerging Infectious Diseases. Nature (2004) 430:242–9. 10.1038/nature02759 PMC7094993 15241422

[29] Lewis K. Platforms for Antibiotic Discovery. Nat Rev Drug Discov (2013) 12:371–87. 10.1038/nrd3975 23629505

[30] Neu HC. The Crisis in Antibiotic Resistance. Science (1992) 257:1064–73. 10.1126/science.257.5073.1064 1509257

[31] Martínez JL. Antibiotics and Antibiotic Resistance Genes in Natural Environments. Science (2008) 321:365–7. 10.1126/science.1159483 18635792

[32] Bush K Courvalin P Dantas G Davies J Eisenstein B Huovinen P. Tackling Antibiotic Resistance. Nat Rev Microbiol (2011) 9:894–6. 10.1038/nrmicro2693 PMC4206945 22048738

[33] Cipolla-Neto J Amaral FGD. Melatonin as a Hormone: New Physiological and Clinical Insights. Endocr Rev (2018) 39:990–1028. 10.1210/er.2018-00084 30215696

[34] Rusanova I Martínez-Ruiz L lorido J Rodríguez-Santana C Guerra-Librero A Acuña-Castroviejo D. Protective Effects of Melatonin on the Skin: Future Perspectives. Int J Mol Sci (2019) 20:4948. 10.3390/ijms20194948 PMC6802208 31597233

[35] Tordjman S Chokron S Delorme R Charrier A Bellissant E Jaafari N. Melatonin: Pharmacology, Functions and Therapeutic Benefits. Curr Neuropharmacol (2017) 15:434–43. 10.2174/1570159x14666161228122115 PMC5405617 28503116

[36] Wang B Wen H Smith W Hao D He B Kong L. Regulation Effects of Melatonin on Bone Marrow Mesenchymal Stem Cell Differentiation. J Cell Physiol (2019) 234:1008–15. 10.1002/jcp.27090 30145787

[37] Hardeland R Cardinali DP Srinivasan V Spence DW Brown GM Pandi-Perumal SR. Melatonin–a Pleiotropic, Orchestrating Regulator Molecule. Prog Neurobiol (2011) 93:350–84. 10.1016/j.pneurobio.2010.12.004 21193011

[38] Pandi-Perumal SR Srinivasan V Maestroni GJ Cardinali DP Poeggeler B Hardeland R. Melatonin: Nature’s Most Versatile Biological Signal? FEBS J (2006) 273:2813–38. 10.1111/j.1742-4658.2006.05322.x 16817850

[39] Reiter RJ. The Pineal and its Hormones in the Control of Reproduction in Mammals. Endocr Rev (1980) 1:109–31. 10.1210/edrv-1-2-109 6263600

[40] Plaimee P Khamphio M Weerapreeyakul N Barusrux S Johns NP. Immunomodulatory Effect of Melatonin in SK-LU-1 Human Lung Adenocarcinoma Cells Co-Cultured With Peripheral Blood Mononuclear Cells. Cell Prolif (2014) 47:406–15. 10.1111/cpr.12119 PMC6496749 25053373

[41] Xia Y Chen S Zeng S Zhao Y Zhu C Deng B. Melatonin in Macrophage Biology: Current Understanding and Future Perspectives. J Pineal Res (2019) 66:e12547. 10.1111/jpi.12547 30597604

[42] Ren W Liu G Chen S Yin J Wang J Tan B. Melatonin Signaling in T Cells: Functions and Applications. J Pineal Res (2017) 62:e12394. 10.1111/jpi.12394 28152213

[43] Tan D-X Hardeland R. Potential Utility of Melatonin in Deadly Infectious Diseases Related to the Overreaction of Innate Immune Response and Destructive Inflammation: Focus on COVID-19. Melatonin Res (2020) 3:120–43. 10.32794/mr11250052

[44] Mauriz JL Collado PS Veneroso C Reiter RJ González-Gallego J. A Review of the Molecular Aspects of Melatonin’s Anti-Inflammatory Actions: Recent Insights and New Perspectives. J Pineal Res (2013) 54:1–14. 10.1111/j.1600-079X.2012.01014.x 22725668

[45] Álvarez-Sánchez N Cruz-Chamorro I Díaz-Sánchez M Sarmiento-Soto H Medrano-Campillo P Martínez-López A. Melatonin Reduces Inflammatory Response in Peripheral T Helper Lymphocytes From Relapsing-Remitting Multiple Sclerosis Patients. J Pineal Res (2017) 63:e12394. 10.1111/jpi.12442 28793364

[46] Hardeland R. Aging, Melatonin, and the Pro- and Anti-Inflammatory Networks. Int J Mol Sci (2019) 20:1223. 10.3390/ijms20051223 PMC6429360 30862067

[47] Yawoot N Govitrapong P Tocharus C. Ischemic Stroke, Obesity, and the Anti-Inflammatory Role of Melatonin. Biofactors (2021) 47:41–58. 10.1002/biof.1690 33135223

[48] Reiter RJ Tan DX Burkhardt S. Reactive Oxygen and Nitrogen Species and Cellular and Organismal Decline: Amelioration With Melatonin. Mech Ageing Dev (2002) 123:1007–19. 10.1016/s0047-6374(01)00384-0 12044950

[49] Reiter RJ Tan DX Mayo JC Sainz RM Leon J Czarnocki Z. Melatonin as an Antioxidant: Biochemical Mechanisms and Pathophysiological Implications in Humans. Acta Biochim Pol (2003) 50:1129–46. 10.18388/abp.2003_3637 14740000

[50] Hardeland R. Antioxidative Protection by Melatonin: Multiplicity of Mechanisms From Radical Detoxification to Radical Avoidance. Endocrine (2005) 27:119–30. 10.1385/endo:27:2:119 16217125

[51] Tian X Wang F Zhang L Ji P Wang J Lv D. Melatonin Promotes the In Vitro Development of Microinjected Pronuclear Mouse Embryos Via Its Anti-Oxidative and Anti-Apoptotic Effects. Int J Mol Sci (2017) 18:988. 10.3390/ijms18050988 PMC5454901 28475125

[52] Hill SM Frasch T Xiang S Yuan L Duplessis T Mao L. Molecular Mechanisms of Melatonin Anticancer Effects. Integr Cancer Ther (2009) 8:337–46. 10.1177/1534735409353332 20050373

[53] Samanta S. Melatonin: An Endogenous Miraculous Indolamine, Fights Against Cancer Progression. J Cancer Res Clin Oncol (2020) 146:1893–922. 10.1007/s00432-020-03292-w PMC11804358 32583237

[54] Tekbas OF Ogur R Korkmaz A Kilic A Reiter RJ. Melatonin as an Antibiotic: New Insights Into the Actions of This Ubiquitous Molecule. J Pineal Res (2008) 44:222–6. 10.1111/j.1600-079X.2007.00516.x 18289175

[55] Lee HY Byeon Y Back K. Melatonin as a Signal Molecule Triggering Defense Responses Against Pathogen Attack in Arabidopsis and Tobacco. J Pineal Res (2014) 57:262–8. 10.1111/jpi.12165 25099383

[56] Bishayi B Adhikary R Nandi A Sultana S. Beneficial Effects of Exogenous Melatonin in Acute Staphylococcus Aureus and Escherichia Coli Infection-Induced Inflammation and Associated Behavioral Response in Mice After Exposure to Short Photoperiod. Inflammation (2016) 39:2072–93. 10.1007/s10753-016-0445-9 27682182

[57] López LC Escames G Tapias V Utrilla P León J Acuña-Castroviejo D. Identification of an Inducible Nitric Oxide Synthase in Diaphragm Mitochondria From Septic Mice: its Relation With Mitochondrial Dysfunction and Prevention by Melatonin. Int J Biochem Cell Biol (2006) 38:267–78. 10.1016/j.biocel.2005.09.008 16223598

[58] Escames G Acuña-Castroviejo D López LC Tan DX Maldonado MD Sánchez-Hidalgo M. Pharmacological Utility of Melatonin in the Treatment of Septic Shock: Experimental and Clinical Evidence. J Pharm Pharmacol (2006) 58:1153–65. 10.1211/jpp.58.9.0001 16945173

[59] Ortiz F García JA Acuña-Castroviejo D Doerrier C López A Venegas C. The Beneficial Effects of Melatonin Against Heart Mitochondrial Impairment During Sepsis: Inhibition of iNOS and Preservation of Nnos. J Pineal Res (2014) 56:71–81. 10.1111/jpi.12099 24117944

[60] Henderson R Kim S Lee E. Use of Melatonin as Adjunctive Therapy in Neonatal Sepsis: A Systematic Review and Meta-Analysis. Complement Ther Med (2018) 39:131–6. 10.1016/j.ctim.2018.06.002 30012383

[61] Srinivasan V Mohamed M Kato H. Melatonin in Bacterial and Viral Infections With Focus on Sepsis: A Review. Recent Pat Endocr Metab Immune Drug Discov (2012) 6:30–9. 10.2174/187221412799015317 22264213

[62] Galley HF Lowes DA Allen L Cameron G Aucott LS Webster NR. Melatonin as a Potential Therapy for Sepsis: A Phase I Dose Escalation Study and an Ex Vivo Whole Blood Model Under Conditions of Sepsis. J Pineal Res (2014) 56:427–38. 10.1111/jpi.12134 PMC4279949 24650045

[63] Hardeland R. Melatonin and Inflammation-Story of a Double-Edged Blade. J Pineal Res (2018) 65:e12525. 10.1111/jpi.12525 30242884

[64] Tan DX Zheng X Kong J Manchester LC Hardeland R Kim SJ. Fundamental Issues Related to the Origin of Melatonin and Melatonin Isomers During Evolution: Relation to Their Biological Functions. Int J Mol Sci (2014) 15:15858–90. 10.3390/ijms150915858 PMC4200856 25207599

[65] Tan DX Hardeland R Back K Manchester LC Alatorre-Jimenez MA Reiter RJ. On the Significance of an Alternate Pathway of Melatonin Synthesis Via 5-Methoxytryptamine: Comparisons Across Species. J Pineal Res (2016) 61:27–40. 10.1111/jpi.12336 27112772

[66] Luo H Schneider K Christensen U Lei Y Herrgard M Palsson B. Microbial Synthesis of Human-Hormone Melatonin at Gram Scales. ACS Synth Biol (2020) 9:1240–5. 10.1021/acssynbio.0c00065 32501000

[67] Tan DX Manchester LC Liu X Rosales-Corral SA Acuna-Castroviejo D Reiter RJ. Mitochondria and Chloroplasts as the Original Sites of Melatonin Synthesis: A Hypothesis Related to Melatonin’s Primary Function and Evolution in Eukaryotes. J Pineal Res (2013) 54:127–38. 10.1111/jpi.12026 23137057

[68] Hardeland R. Melatonin in the Evolution of Plants and Other Phototrophs. Melatonin Res (2019) 2:10–36. 10.32794/mr11250029

[69] Claustrat B Brun J Chazot G. The Basic Physiology and Pathophysiology of Melatonin. Sleep Med Rev (2005) 9:11–24. 10.1016/j.smrv.2004.08.001 15649735

[70] Hardeland R Madrid JA Tan DX Reiter RJ. Melatonin, the Circadian Multioscillator System and Health: The Need for Detailed Analyses of Peripheral Melatonin Signaling. J Pineal Res (2012) 52:139–66. 10.1111/j.1600-079X.2011.00934.x 22034907

[71] Zisapel N. New Perspectives on the Role of Melatonin in Human Sleep, Circadian Rhythms and Their Regulation. Br J Pharmacol (2018) 175:3190–9. 10.1111/bph.14116 PMC6057895 29318587

[72] Scheer FA Van Montfrans GA van Someren EJ Mairuhu G Buijs RM. Daily Nighttime Melatonin Reduces Blood Pressure in Male Patients With Essential Hypertension. Hypertension (2004) 43:192–7. 10.1161/01.HYP.0000113293.15186.3b 14732734

[73] Rodríguez C Puente-Moncada N. Regulation of Cancer Cell Glucose Metabolism is Determinant for Cancer Cell Fate After Melatonin Administration. J Cell Physiol (2021) 236:27–40. 10.1002/jcp.29886 32725819

[74] Dollins AB Zhdanova IV Wurtman RJ Lynch HJ Deng MH. Effect of Inducing Nocturnal Serum Melatonin Concentrations in Daytime on Sleep, Mood, Body Temperature, and Performance. Proc Natl Acad Sci U S A (1994) 91:1824–8. 10.1073/pnas.91.5.1824 PMC43256 8127888

[75] Reiter RJ Rosales-Corral SA Tan DX Acuna-Castroviejo D Qin L Yang SF. Melatonin, a Full Service Anti-Cancer Agent: Inhibition of Initiation, Progression and Metastasis. Int J Mol Sci (2017) 18:843. 10.3390/ijms18040843 PMC5412427 28420185

[76] Chitimus DM Popescu MR Voiculescu SE Panaitescu AM Pavel B Zagrean L. Melatonin’s Impact on Antioxidative and Anti-Inflammatory Reprogramming in Homeostasis and Disease. Biomolecules (2020) 10:1211. 10.3390/biom10091211 PMC7563541 32825327

[77] Ma Q Reiter RJ Chen Y. Role of Melatonin in Controlling Angiogenesis Under Physiological and Pathological Conditions. Angiogenesis (2020) 23:91–104. 10.1007/s10456-019-09689-7 31650428

[78] Claustrat B Leston J. Melatonin: Physiological Effects in Humans. Neurochirurgie (2015) 61:77–84. 10.1016/j.neuchi.2015.03.002 25908646

[79] Mistraletti G Umbrello M Sabbatini G Miori S Taverna M Cerri B. Melatonin Reduces the Need for Sedation in ICU Patients: A Randomized Controlled Trial. Minerva Anestesiol (2015) 81:1298–310. 25969139

[80] Mistraletti G Sabbatini G Taverna M Figini MA Umbrello M Magni P. Pharmacokinetics of Orally Administered Melatonin in Critically Ill Patients. J Pineal Res (2010) 48:142–7. 10.1111/j.1600-079X.2009.00737.x 20070489

[81] Zhang R Wang X Ni L Di X Ma B Niu S. Covid-19: Melatonin as a Potential Adjuvant Treatment. Life Sci (2020) 250:117583. 10.1016/j.lfs.2020.117583 32217117 PMC7102583

[82] Nordlund JJ Lerner AB. The Effects of Oral Melatonin on Skin Color and on the Release of Pituitary Hormones. J Clin Endocrinol Metab (1977) 45:768–74. 10.1210/jcem-45-4-768 914981

[83] Barchas J DaCosta F Spector S. Acute Pharmacology of Melatonin. Nature (1967) 214:919–20. 10.1038/214919a0 6054984

[84] Sugden D. Psychopharmacological Effects of Melatonin in Mouse and Rat. J Pharmacol Exp Ther (1983) 227:587–91. 10.1016/0160-5402(83)90069-4 6655558

[85] Weishaupt JH Bartels C Pölking E Dietrich J Rohde G Poeggeler B. Reduced Oxidative Damage in ALS by High-Dose Enteral Melatonin Treatment. J Pineal Res (2006) 41:313–23. 10.1111/j.1600-079X.2006.00377.x 17014688

[86] Wasdell MB Jan JE Bomben MM Freeman RD Rietveld WJ Tai J. A Randomized, Placebo-Controlled Trial of Controlled Release Melatonin Treatment of Delayed Sleep Phase Syndrome and Impaired Sleep Maintenance in Children With Neurodevelopmental Disabilities. J Pineal Res (2008) 44:57–64. 10.1111/j.1600-079X.2007.00528.x 18078449

[87] Andersen LP Gögenur I Rosenberg J Reiter RJ. The Safety of Melatonin in Humans. Clin Drug Investig (2016) 36:169–75. 10.1007/s40261-015-0368-5 26692007

[88] Ferlazzo N Andolina G Cannata A Costanzo MG Rizzo V Currò M. Is Melatonin the Cornucopia of the 21st Century? Antioxidants (Basel) (2020) 9:1088. 10.3390/antiox9111088 PMC7694322 33167396

[89] Besag FMC Vasey MJ Lao KSJ Wong ICK. Adverse Events Associated With Melatonin for the Treatment of Primary or Secondary Sleep Disorders: A Systematic Review. CNS Drugs (2019) 33:1167–86. 10.1007/s40263-019-00680-w 31722088

[90] Ozturk AI Yilmaz O Kirbag S Arslan M. Antimicrobial and Biological Effects of Ipemphos and Amphos on Bacterial and Yeast Strains. Cell Biochem Funct (2000) 18:117–26. 10.1002/(sici)1099-0844(200006)18:2<117::aid-cbf863>3.0.co;2-1 10814970

[91] Wang HX Liu F Ng TB. Examination of Pineal Indoles and 6-methoxy-2-benzoxazolinone for Antioxidant and Antimicrobial Effects. Comp Biochem Physiol C Toxicol Pharmacol (2001) 130:379–88. 10.1016/S1532-0456(01)00264-2 11701394

[92] Wiid I Hoalvan HE Hon D Lombard C Van HP. Potentiation of Isoniazid Activity Against Mycobacterium Tuberculosis by Melatonin. Antimicrob Agents Chemother (1999) 43:975–7. 10.1128/AAC.43.4.975 PMC89241 10103215

[93] Abbas M Paul M Huttner A. New and Improved? A Review of Novel Antibiotics for Gram-Positive Bacteria. Clin Microbiol Infect (2017) 23:697–703. 10.1016/j.cmi.2017.06.010 28642145

[94] Woodford N Livermore DM. Infections Caused by Gram-Positive Bacteria: A Review of the Global Challenge. J Infect (2009) 59 Suppl 1:S4–16. 10.1016/s0163-4453(09)60003-7 19766888

[95] Atroshi F Rizzo A Westermarck T Ali-vehmas T. Effects of Tamoxifen, Melatonin, Coenzyme Q10, and L-carnitine Supplementation on Bacterial Growth in the Presence of Mycotoxins. Pharmacol Res (1998) 38:289–95. 10.1006/phrs.1998.0363 9774492

[96] Masadeh MM Alzoubi KH Al-Azzam SI Khabour OF Al-Buhairan AM. Ciprofloxacin-Induced Antibacterial Activity Is Atteneuated by Pretreatment With Antioxidant Agents. Pathogens (2016) 5:28. 10.3390/pathogens5010028 PMC4810149 27005666

[97] Thi MTT Wibowo D Rehm BHA. Pseudomonas Aeruginosa Biofilms. Int J Mol Sci (2020) 21:8671. 10.3390/ijms21228671 PMC7698413 33212950

[98] Chen W. Host Innate Immune Responses to Acinetobacter Baumannii Infection. Front Cell Infect Microbiol (2020) 10:486. 10.3389/fcimb.2020.00486 33042864 PMC7521131

[99] de Bentzmann S Plésiat P. The Pseudomonas Aeruginosa Opportunistic Pathogen and Human Infections. Environ Microbiol (2011) 13:1655–65. 10.1111/j.1462-2920.2011.02469.x 21450006

[100] Redfern J Enright MC. Further Understanding of Pseudomonas Aeruginosa’s Ability to Horizontally Acquire Virulence: Possible Intervention Strategies. Expert Rev Anti Infect Ther (2020) 18:539–49. 10.1080/14787210.2020.1751610 32249619

[101] Augustin P Tran-Dinh A Valin N Desmard M Crevecoeur MA Muller-Serieys C. Pseudomonas Aeruginosa Post-Operative Peritonitis: Clinical Features, Risk Factors, and Prognosis. Surg Infect (Larchmt) (2013) 14:297–303. 10.1089/sur.2012.084 23672242

[102] Brandenburg KS Weaver AJ Jr. Qian L You T Chen P Karna SLR. Development of Pseudomonas Aeruginosa Biofilms in Partial-Thickness Burn Wounds Using a Sprague-Dawley Rat Model. J Burn Care Res (2019) 40:44–57. 10.1093/jbcr/iry043 30137429 PMC6300396

[103] Yoshida S Suzuki K Suzuki A Okada H Niwa T Kobayashi R. Risk Factors for the Failure of Treatment of Pseudomonas Aeruginosa Bacteremia in Critically Ill Patients. Pharmazie (2017) 72:428–32. 10.1691/ph.2017.7453 29441942

[104] Britt NS Ritchie DJ Kollef MH Burnham CA Durkin MJ Hampton NB. Importance of Site of Infection and Antibiotic Selection in the Treatment of Carbapenem-Resistant Pseudomonas Aeruginosa Sepsis. Antimicrob Agents Chemother (2018) 62:e02400–17. 10.1128/aac.02400-17 PMC5913923 29378722

[105] Robertson GT Doyle TB Du Q Duncan L Mdluli KE Lynch AS. A Novel Indole Compound That Inhibits Pseudomonas Aeruginosa Growth by Targeting MreB is a Substrate for Mexab-Oprm. J Bacteriol (2007) 189:6870–81. 10.1128/jb.00805-07 PMC2045200 17644596

[106] Wareth G Neubauer H Sprague LD. Acinetobacter Baumannii - a Neglected Pathogen in Veterinary and Environmental Health in Germany. Vet Res Commun (2019) 43:1–6. 10.1007/s11259-018-9742-0 30591981

[107] Amaya-Villar R Garnacho-Montero J. How Should We Treat Acinetobacter Pneumonia? Curr Opin Crit Care (2019) 25:465–72. 10.1097/mcc.0000000000000649 31335380

[108] Xiao J Zhang C Ye S. Acinetobacter Baumannii Meningitis in Children: A Case Series and Literature Review. Infection (2019) 47:643–9. 10.1007/s15010-018-1234-1 30328074

[109] Chusri S Chongsuvivatwong V Silpapojakul K Singkhamanan K Hortiwakul T Charernmak B. Clinical Characteristics and Outcomes of Community and Hospital-Acquired Acinetobacter Baumannii Bacteremia. J Microbiol Immunol Infect (2019) 52:796–806. 10.1016/j.jmii.2019.03.004 31031096

[110] Shi J Sun T Cui Y Wang C Wang F Zhou Y. Multidrug Resistant and Extensively Drug Resistant Acinetobacter Baumannii Hospital Infection Associated With High Mortality: A Retrospective Study in the Pediatric Intensive Care Unit. BMC Infect Dis (2020) 20:597. 10.1186/s12879-020-05321-y 32787942 PMC7422664

[111] Fernández-García L Fernandez-Cuenca F Blasco L López-Rojas R Ambroa A Lopez M. Relationship Between Tolerance and Persistence Mechanisms in Acinetobacter Baumannii Strains With AbkAB Toxin-Antitoxin System. Antimicrob Agents Chemother (2018) 62:e00250–00218. 10.1128/aac.00250-18 PMC5923160 29463538

[112] Uberos J Augustin C Liébana J Molina A Muñoz-Hoyos A. Comparative Study of the Influence of Melatonin and Vitamin E on the Surface Characteristics of Escherichia Coli. Lett Appl Microbiol (2001) 32:303–6. 10.1046/j.1472-765x.2001.00908.x 11328494

[113] Uberos J Molina A Liébana J Augustin MC Muñoz A. The Influence of Different Concentrations of Melatonin on the Cell Surface Hydrophobic Characteristics of Neisseria Meningitidis. Lett Appl Microbiol (2000) 31:294–8. 10.1046/j.1472-765x.2000.00813.x 11068910

[114] Gagneux S. Ecology and Evolution of Mycobacterium Tuberculosis. Nat Rev Microbiol (2018) 16:202–13. 10.1038/nrmicro.2018.8 29456241

[115] Pavlik A Yayo I Parmova W Melicharek I Hanzlikova I Svejcova M. Mycobacterium Tuberculosis in Animal and Human Population in Six Central European Countries During 1990-1999. Vet Med (2003) 48:8–89. 10.17221/5754-VETMED

[116] Chen Y Chao Y Deng Q Liu T Xiang J Chen J. Potential Challenges to the Stop Tb Plan for Humans in China; Cattle Maintain M. Bovis, and M. Tuberculosis. Tuberculosis (Edinb) (2009) 89:95–100. 10.1016/j.tube.2008.07.003 19056318

[117] Sarathy JP Via LE Weiner D Blanc L Boshoff H Eugenin EA. Extreme Drug Tolerance of Mycobacterium Tuberculosis in Caseum. Antimicrob Agents Chemother (2018) 62:e02266–02217. 10.1128/aac.02266-17 PMC5786764 29203492

[118] Jasim TM Alabbassi MG Almuqdadi SF Kamel H Kamel J. Anti-Bacterial Properties of Melatonin Against Mycobacterium Tuberculosis In Vitro. Iraqi J Pharm Sci (2010) 19:59–63.

[119] Chen X Sun C Laborda P Zhao Y Palmer I Fu ZQ. Melatonin Treatment Inhibits the Growth of Xanthomonas Oryzae Pv. Oryzae. Front Microbiol (2018) 9:2280. 10.3389/fmicb.2018.02280 30337911 PMC6180160

[120] Chen X Sun C Laborda P He Y Zhao Y Li Z. Melatonin Treatments Reduce the Pathogenicity and Inhibit the Growth of Xanthomonas Oryzae Pv. Oryzicola. Plant Pathol (2019) 68:288–96. 10.1111/ppa.12954

[121] Ward CG Bullen JJ Rogers HJ. Iron and Infection: New Developments and Their Implications. J Trauma (1996) 41:356–64. 10.1097/00005373-199608000-00030 8760553

[122] Gulcin I Buyukokuroglu ME Kufrevioglu OI. Metal Chelating and Hydrogen Peroxide Scavenging Effects of Melatonin. J Pineal Res (2003) 34:278–81. 10.1034/j.1600-079X.2003.00042.x 12662350

[123] Rothfield L Horecker BL. The Role of Cell-Wall Lipid in the Biosynthesis of Bacterial Lipopolysaccharide. Proc Natl Acad Sci U S A (1964) 52:939–46. 10.1073/pnas.52.4.939 PMC300376 14224398

[124] Hebeler BH Chatterjee AN Young FE. Regulation of the Bacterial Cell Wall: Effect of Antibiotics on Lipid Biosynthesis. Antimicrob Agents Chemother (1973) 4:346–53. 10.1128/AAC.4.3.346 PMC444555 4758838

[125] Dauchy RT Dauchy EM Davidson LK Krause JA Lynch DT Tirrell PC. Inhibition of Fatty Acid Transport and Proliferative Activity in Tissue-Isolated Human Squamous Cell Cancer Xenografts Perfused in Situ With Melatonin or Eicosapentaenoic or Conjugated Linoleic Acids. Comp Med (2007) 57:377–82. 10.1023/A:1010330504668 17803052

[126] Liu Y Jia Y Yang K Tong Z Shi J Li R. Melatonin Overcomes MCR-Mediated Colistin Resistance in Gram-negative Pathogens. Theranostics (2020) 10:10697–711. 10.7150/thno.45951 PMC7482817 32929375

[127] Gerber J Lotz M Ebert S Kiel S Huether G Kuhnt U. Melatonin is Neuroprotective in Experimental Streptococcus Pneumoniae Meningitis. J Infect Dis (2005) 191:783–90. 10.1086/427816 15688296

[128] Sener G Tuğtepe H Velioğlu-Oğünç A Cetinel S Gedik N Yeğen BC. Melatonin Prevents Neutrophil-Mediated Oxidative Injury in Escherichia Coli-Induced Pyelonephritis in Rats. J Pineal Res (2006) 41:220–7. 10.1111/j.1600-079X.2006.00357.x 16948782

[129] Luo J Song J Zhang H Zhang F Liu H Li L. Melatonin Mediated Foxp3-Downregulation Decreases Cytokines Production Via the TLR2 and TLR4 Pathways in H. Pylori Infected Mice. Int Immunopharmacol (2018) 64:116–22. 10.1016/j.intimp.2018.08.034 30173051

[130] Wu UI Mai FD Sheu JN Chen LY Liu YT Huang HC. Melatonin Inhibits Microglial Activation, Reduces Pro-Inflammatory Cytokine Levels, and Rescues Hippocampal Neurons of Adult Rats With Acute Klebsiella Pneumoniae Meningitis. J Pineal Res (2011) 50:159–70. 10.1111/j.1600-079X.2010.00825.x 21062353

[131] Carrillo-Vico A Lardone PJ Naji L Fernandez-Santos JM Martin-Lacave I Guerrero JM. Beneficial Pleiotropic Actions of Melatonin in an Experimental Model of Septic Shock in Mice: Regulation of Pro-/Anti-Inflammatory Cytokine Network, Protection Against Oxidative Damage and Anti-Apoptotic Effects. J Pineal Res (2005) 39:400–8. 10.1111/j.1600-079X.2005.00265.x 16207296

[132] Maestroni GJ. Melatonin as a Therapeutic Agent in Experimental Endotoxic Shock. J Pineal Res (1996) 20:84–9. 10.1111/j.1600-079X.1996.tb00244.x 8815192

[133] de Jong NWM van Kessel KPM van Strijp JAG. Immune Evasion by Staphylococcus Aureus. Microbiol Spectr (2019) 7:GPP3-0061-201. 10.1128/microbiolspec.GPP3-0061-2019 PMC11590434 30927347

[134] Smith TC Wardyn SE. Human Infections With Staphylococcus Aureus CC398. Curr Environ Health Rep (2015) 2:41–51. 10.1007/s40572-014-0034-8 26231241

[135] Reddinger RM Luke-Marshall NR Sauberan SL Hakansson AP. Streptococcus Pneumoniae Modulates Staphylococcus Aureus Biofilm Dispersion and the Transition From Colonization to Invasive Disease. mBio (2018) 9:e02089–02017. 10.1128/mBio.02089-17 PMC5760742 29317512

[136] Zhao W Pan F Wang B Wang C Sun Y Zhang T. Epidemiology Characteristics of Streptococcus Pneumoniae From Children With Pneumonia in Shanghai: A Retrospective Study. Front Cell Infect Microbiol (2019) 9:258. 10.3389/fcimb.2019.00258 31380301 PMC6657011

[137] Yau B Hunt NH Mitchell AJ Too LK. Blood–Brain Barrier Pathology and CNS Outcomes in Streptococcus Pneumoniae Meningitis. Int J Mol Sci (2018) 19:3555. 10.3390/ijms19113555 PMC6275034 30423890

[138] Priftis KN Litt D Manglani S Anthracopoulos MB Thickett K Tzanakaki G. Bacterial Bronchitis Caused by Streptococcus Pneumoniae and Nontypable Haemophilus Influenzae in Children: The Impact of Vaccination. Chest (2013) 143:152–7. 10.1378/chest.12-0623 22911476

[139] Spreer A Gerber J Baake D Hanssen M Huether G Nau R. Antiinflammatory But No Neuroprotective Effects of Melatonin Under Clinical Treatment Conditions in Rabbit Models of Bacterial Meningitis. J Neurosci Res (2006) 84:1575–9. 10.1002/jnr.21055 16998917

[140] Mueller M Tainter CR. Escherichia Coli. In: Statpearls. Treasure Island (FL: StatPearls Publishing (2020). Copyright © 2020, StatPearls Publishing LLC.

[141] Köhler CD Dobrindt U. What Defines Extraintestinal Pathogenic Escherichia Coli? Int J Med Microbiol (2011) 301:642–7. 10.1016/j.ijmm.2011.09.006 21982038

[142] Madhavan TP Sakellaris H. Colonization Factors of Enterotoxigenic Escherichia Coli. Adv Appl Microbiol (2015) 90:155–97. 10.1016/bs.aambs.2014.09.003 25596032

[143] Bohnlein C Kabisch J Meske D Franz CM Pichner R. Fitness of Enterohemorrhagic Escherichia Coli (EHEC)/Enteroaggregative E. Coli O104:H4 in Comparison to That of EHEC O157: Survival Studies in Food and In Vitro. Appl Environ Microbiol (2016) 82:6326–34. 10.1128/aem.01796-16 PMC5066349 27542931

[144] Hu J Torres AG. Enteropathogenic Escherichia Coli: Foe or Innocent Bystander? Clin Microbiol Infect (2015) 21:729–34. 10.1016/j.cmi.2015.01.015 PMC4497942 25726041

[145] Vijay D Dhaka P Vergis J Negi M Mohan V Kumar M. Characterization and Biofilm Forming Ability of Diarrhoeagenic Enteroaggregative Escherichia Coli Isolates Recovered From Human Infants and Young Animals. Comp Immunol Microbiol Infect Dis (2015) 38:21–31. 10.1016/j.cimid.2014.11.004 25529123

[146] Ramya Raghavan P Purushothaman SA Ramanathan T Roy S. Diarrhegenic Escherichia Coli Replaces Shigella Spp. As the Predominant Bacteria Causing Childhood Diarrhea in Andaman and Nicobar Islands, India. Am J Trop Med Hyg (2018) 98:814–5. 10.4269/ajtmh.17-0327 PMC5930883 29363445

[147] Khwaja J. Bilateral Adrenal Hemorrhage in the Background of Escherichia Coli Sepsis: A Case Report. J Med Case Rep (2017) 11:72. 10.1186/s13256-017-1236-0 28302165 PMC5356297

[148] Paulose JK Wright JM Patel AG Cassone VM. Human Gut Bacteria Are Sensitive to Melatonin and Express Endogenous Circadian Rhythmicity. PloS One (2016) 11:e0146643. 10.1371/journal.pone.0146643 26751389 PMC4709092

[149] Dunne C Dolan B Clyne M. Factors That Mediate Colonization of the Human Stomach by Helicobacter Pylori. World J Gastroenterol (2014) 20:5610–24. 10.3748/wjg.v20.i19.5610 PMC4024769 24914320

[150] Yang L Zhang J Xu J Wei X Yang J Liu Y. Helicobacter Pylori Infection Aggravates Dysbiosis of Gut Microbiome in Children With Gastritis. Front Cell Infect Microbiol (2019) 9:375. 10.3389/fcimb.2019.00375 31781514 PMC6859803

[151] Lanas A Dumonceau JM Hunt RH Fujishiro M Scheiman JM Gralnek IM. Non-Variceal Upper Gastrointestinal Bleeding. Nat Rev Dis Primers (2018) 4:18020. 10.1038/nrdp.2018.20 29671413

[152] Kim SS Ruiz VE Carroll JD Moss SF. Helicobacter Pylori in the Pathogenesis of Gastric Cancer and Gastric Lymphoma. Cancer Lett (2011) 305:228–38. 10.1016/j.canlet.2010.07.014 PMC2980557 20692762

[153] Chojnacki C Poplawski T Blasiak J Chojnacki J Reiter RJ Klupinska G. Expression of Melatonin Synthesizing Enzymes in Helicobacter Pylori Infected Gastric Mucosa. BioMed Res Int (2013) 2013:845032. 10.1155/2013/845032 23936850 PMC3722974

[154] Malinovskaia NK Rapoport SI Zhernakova NI Rybnikova SN Postnikova LI Parkhomenko IE. [Antihelicobacter Effects of Melatonin]. Klin Med (Mosk) (2007) 85:40–3. 17523403

[155] Celinski K Konturek PC Konturek SJ Slomka M Cichoz-Lach H Brzozowski T. Effects of Melatonin and Tryptophan on Healing of Gastric and Duodenal Ulcers With Helicobacter Pylori Infection in Humans. J Physiol Pharmacol (2011) 62:521–6. 22204799

[156] Osadchuk MA Sibriaev AA Kireeva NV Kvetnoi IM. [The Influence of Melatonin Included in the Combined Treatment of Antichelicobaterial Therapy on Immunohistochemical Characteristics of Gastric Epitheliocytes From Patients With Duodenal Ulcer]. Klin Med (Mosk) (2012) 90:48–52. 23516871

[157] Ikeda M Mizoguchi M Oshida Y Tatsuno K Saito R Okazaki M. Clinical and Microbiological Characteristics and Occurrence of Klebsiella Pneumoniae Infection in Japan. Int J Gen Med (2018) 11:293–9. 10.2147/ijgm.s166940 PMC6049057 30034248

[158] Bueno MG Iovine RO Torres LN Catao-Dias JL Pissinatti A Kierulff MC. Pneumonia and Bacteremia in a Golden-Headed Lion Tamarin (Leontopithecus Chrysomelas) Caused by Klebsiella Pneumoniae Subsp. Pneumoniae During a Translocation Program of Free-Ranging Animals in Brazil. J Vet Diagn Invest (2015) 27:387–91. 10.1177/1040638715584792 25943130

[159] Bidewell CA Williamson SM Rogers J Tang Y Ellis RJ Petrovska L. Emergence of Klebsiella Pneumoniae Subspecies Pneumoniae as a Cause of Septicaemia in Pigs in England. PLoS One (2018) 13:e0191958. 10.1371/journal.pone.0191958 29470491 PMC5823397

[160] Namikawa H Yamada K Sakiyama A Imoto W Yamairi K Shibata W. Clinical Characteristics of Bacteremia Caused by Hypermucoviscous Klebsiella Pneumoniae at a Tertiary Hospital. Diagn Microbiol Infect Dis (2019) 95:84–8. 10.1016/j.diagmicrobio.2019.04.008 31256940

[161] Carrie C Walewski V Levy C Alexandre C Baleine J Charreton C. Klebsiella Pneumoniae and Klebsiella Oxytoca Meningitis in Infants. Epidemiological and Clinical Features. Arch Pediatr (2019) 26:12–5. 10.1016/j.arcped.2018.09.013 30558858

[162] Dhruve MJ Bargman JM. Klebsiella Pneumoniae Renal Abscess and Peritonitis in a Peritoneal Dialysis Patient: A Novel Route of Infection. Perit Dial Int (2017) 37:654–6. 10.3747/pdi.2017.00094 29123004

[163] Korde JP Srivastava RS Mishra SC Sharma AK. Time Dependent Immunomodulatory Response of Exogenous Melatonin to Killed Pasteurella Multocida (P52 Strain) Vaccine in Albino Rats. Indian J Physiol Pharmacol (2005) 49:227–35. 16170993

[164] Esquifino AI Pandi-Perumal SR Cardinali DP. Circadian Organization of the Immune Response: A Role for Melatonin. Clin Appl Immunol Rev (2004) 4:423–33. 10.1016/j.cair.2004.08.002

[165] Colunga Biancatelli RML Berrill M Mohammed YH Marik PE. Melatonin for the Treatment of Sepsis: The Scientific Rationale. J Thorac Dis (2020) 12:S54–s65. 10.21037/jtd.2019.12.85 32148926 PMC7024751

[166] Zhen G Liang W Jia H Zheng X. Melatonin Relieves Sepsis-Induced Myocardial Injury Via Regulating JAK2/STAT3 Signaling Pathway. Minerva Med (2020). 10.23736/s0026-4806.20.06626-4 32683850

[167] Petronilho F Giustina AD Nascimento DZ Zarbato GF Vieira AA Florentino D. Obesity Exacerbates Sepsis-Induced Oxidative Damage in Organs. Inflammation (2016) 39:2062–71. 10.1007/s10753-016-0444-x 27645696

[168] Mantzarlis K Tsolaki V. Role of Oxidative Stress and Mitochondrial Dysfunction in Sepsis and Potential Therapies. Oxid Med Cell Longev (2017) 2017:5985209. 10.1155/2017/5985209 28904739 PMC5585571

[169] Tan DX Manchester LC Terron MP Flores LJ Reiter RJ. One Molecule, Many Derivatives: A Never-Ending Interaction of Melatonin With Reactive Oxygen and Nitrogen Species? J Pineal Res (2007) 42:28–42. 10.1111/j.1600-079X.2006.00407.x 17198536

[170] Chen Y Qing W Sun M Lv L Guo D Jiang Y. Melatonin Protects Hepatocytes Against Bile Acid-Induced Mitochondrial Oxidative Stress Via the AMPK-SIRT3-SOD2 Pathway. Free Radic Res (2015) 49:1275–84. 10.3109/10715762.2015.1067806 26118716

[171] Pi H Xu S Reiter RJ Guo P Zhang L Li Y. Sirt3-SOD2-mROS-dependent Autophagy in Cadmium-Induced Hepatotoxicity and Salvage by Melatonin. Autophagy (2015) 11:1037–51. 10.1080/15548627.2015.1052208 PMC4590599 26120888

[172] Zhai M Li B Duan W Jing L Zhang B Zhang M Jing L Zhang B Zhang M. Melatonin Ameliorates Myocardial Ischemia Reperfusion Injury Through SIRT3-Dependent Regulation of Oxidative Stress and Apoptosis. (2017) 63:e12419. 10.1111/jpi.12419 28500761

[173] Zhang J Wang L Xie W Hu S Zhou H Zhu P. Melatonin Attenuates ER Stress and Mitochondrial Damage in Septic Cardiomyopathy: A New Mechanism Involving BAP31 Upregulation and MAPK-ERK Pathway. J Cell Physiol (2020) 235:2847–56. 10.1002/jcp.29190 31535369

[174] Kirkebøen KA Strand OA. The Role of Nitric Oxide in Sepsis–an Overview. Acta Anaesthesiol Scand (1999) 43:275–88. 10.1034/j.1399-6576.1999.430307.x 10081533

[175] Escames G León J Macías M Khaldy H Acuña-Castroviejo D. Melatonin Counteracts Lipopolysaccharide-Induced Expression and Activity of Mitochondrial Nitric Oxide Synthase in Rats. FASEB J (2003) 17:932–4. 10.1096/fj.02-0692fje 12670878

[176] Crespo E Macías M Pozo D Escames G Martín M Vives F. Melatonin Inhibits Expression of the Inducible NO Synthase II in Liver and Lung and Prevents Endotoxemia in Lipopolysaccharide-Induced Multiple Organ Dysfunction Syndrome in Rats. FASEB J (1999) 13:1537–46. 10.1096/fasebj.13.12.1537 10463945

[177] Srinivasan V Pandi-Perumal SR Spence DW Kato H Cardinali DP. Melatonin in Septic Shock: Some Recent Concepts. J Crit Care (2010) 25:656.e651–656. 10.1016/j.jcrc.2010.03.006 20435434

[178] Zhou L Zhao D An H Zhang H Jiang C Yang B. Melatonin Prevents Lung Injury Induced by Hepatic Ischemia-Reperfusion Through Anti-Inflammatory and Anti-Apoptosis Effects. Int Immunopharmacol (2015) 29:462–7. 10.1016/j.intimp.2015.10.012 26490220

[179] Tan DX Hardeland R. Targeting Host Defense System and Rescuing Compromised Mitochondria to Increase Tolerance Against Pathogens by Melatonin may Impact Outcome of Deadly Virus Infection Pertinent to COVID-19. Molecules (2020) 25:4410. 10.3390/molecules25194410 PMC7582936 32992875

[180] Yasmin F Sutradhar S Das P Mukherjee S. Gut Melatonin: A Potent Candidate in the Diversified Journey of Melatonin Research. Gen Comp Endocrinol (2020) 303:113693. 10.1016/j.ygcen.2020.113693 33309697

[181] Ma N Zhang J Reiter RJ Ma X. Melatonin Mediates Mucosal Immune Cells, Microbial Metabolism, and Rhythm Crosstalk: A Therapeutic Target to Reduce Intestinal Inflammation. Med Res Rev (2020) 40:606–32. 10.1002/med.21628 31420885

[182] Xu P Wang J Hong F Wang S Jin X Xue T. Melatonin Prevents Obesity Through Modulation of Gut Microbiota in Mice. J Pineal Res (2017) 62:e12399. 10.1111/jpi.12399 28199741

[183] Yin J Li Y Han H Chen S Gao J Liu G. Melatonin Reprogramming of Gut Microbiota Improves Lipid Dysmetabolism in High-Fat Diet-Fed Mice. J Pineal Res (2018) 65:e12524. 10.1111/jpi.12524 30230594

[184] Yin J Li Y Han H Ma J Liu G Wu X. Administration of Exogenous Melatonin Improves the Diurnal Rhythms of the Gut Microbiota in Mice Fed a High-Fat Diet. mSystems (2020) 5:e00002–20. 10.1128/mSystems.00002-20 PMC7253360 32430404

[185] Hong F Pan S Xu P Xue T Wang J Guo Y. Melatonin Orchestrates Lipid Homeostasis Through the Hepatointestinal Circadian Clock and Microbiota During Constant Light Exposure. Cells (2020) 9:489. 10.3390/cells9020489 PMC7072737 32093272

[186] Lv WJ Liu C. Melatonin Alleviates Neuroinflammation and Metabolic Disorder in DSS-Induced Depression Rats. Oxid Med Cell Longev (2020) 2020:1241894. 10.1155/2020/1241894 32802257 PMC7415091

[187] Zhang H Yan A Liu X Ma Y Zhao F Wang M. Melatonin Ameliorates Ochratoxin A Induced Liver Inflammation, Oxidative Stress and Mitophagy in Mice Involving in Intestinal Microbiota and Restoring the Intestinal Barrier Function. J Hazard Mater (2020) 407:124489. 10.1016/j.jhazmat.2020.124489 33359973

[188] Xu L Zhang W Kwak M Zhang L Lee PCW Jin JO. Protective Effect of Melatonin Against Polymicrobial Sepsis Is Mediated by the Anti-Bacterial Effect of Neutrophils. Front Immunol (2019) 10:1371. 10.3389/fimmu.2019.01371 31275316 PMC6593141

[189] Fink T Glas M Wolf A Kleber A Reus E Wolff M. Melatonin Receptors Mediate Improvements of Survival in a Model of Polymicrobial Sepsis. Crit Care Med (2014) 42:e22–31. 10.1097/CCM.0b013e3182a63e2b 24145838

[190] Zhang Y Li X Grailer JJ Wang N Wang M Yao J. Melatonin Alleviates Acute Lung Injury Through Inhibiting the NLRP3 Inflammasome. J Pineal Res (2016) 60:405–14. 10.1111/jpi.12322 26888116

[191] Lowes DA Almawash AM Webster NR Reid VL Galley HF. Melatonin and Structurally Similar Compounds Have Differing Effects on Inflammation and Mitochondrial Function in Endothelial Cells Under Conditions Mimicking Sepsis. Br J Anaesth (2011) 107:193–201. 10.1093/bja/aer149 21659405

[192] Shang Y Xu SP Wu Y Jiang YX Wu ZY Yuan SY. Melatonin Reduces Acute Lung Injury in Endotoxemic Rats. Chin Med J (Engl) (2009) 122:1388–93. 10.3760/cma.j.issn.0366-6999.2009.12.006 19567158

[193] Titheradge MA. Nitric Oxide in Septic Shock. Biochim Biophys Acta (1999) 1411:437–55. 10.1016/s0005-2728(99)00031-6 10320674

[194] Acuña-Castroviejo D Escames G López LC Hitos AB León J. Melatonin and Nitric Oxide: Two Required Antagonists for Mitochondrial Homeostasis. Endocrine (2005) 27:159–68. 10.1385/endo:27:2:159 16217129

[195] Deng SL Zhang BL Reiter RJ Liu YX. Melatonin Ameliorates Inflammation and Oxidative Stress by Suppressing the P38mapk Signaling Pathway in LPS-Induced Sheep Orchitis. Antioxidants (Basel) (2020) 9:1277. 10.3390/antiox9121277 PMC7765110 33327643

[196] Choi EY Jin JY Lee JY Choi JI Choi IS Kim SJ. Melatonin Inhibits Prevotella Intermedia Lipopolysaccharide-Induced Production of Nitric Oxide and Interleukin-6 in Murine Macrophages by Suppressing NF-kappaB and STAT1 Activity. J Pineal Res (2011) 50:197–206. 10.1111/j.1600-079X.2010.00829.x 21158907

[197] Tamura EK Cecon E Monteiro AW Silva CL Markus RP. Melatonin Inhibits LPS-Induced NO Production in Rat Endothelial Cells. J Pineal Res (2009) 46:268–74. 10.1111/j.1600-079X.2008.00657.x 19215575

[198] Yu GM Kubota H Okita M Maeda T. The Anti-Inflammatory and Antioxidant Effects of Melatonin on LPS-Stimulated Bovine Mammary Epithelial Cells. PLoS One (2017) 12:e0178525. 10.1371/journal.pone.0178525 28542575 PMC5444821

[199] Lee SJ Lee HJ Jung YH Kim JS Choi SH Han HJ. Melatonin Inhibits Apoptotic Cell Death Induced by Vibrio Vulnificus VvhA Via Melatonin Receptor 2 Coupling With NCF-1. Cell Death Dis (2018) 9:48. 10.1038/s41419-017-0083-7 29352110 PMC5833450

[200] Lee YM Park JP Jung YH Lee HJ Kim JS Choi GE. Melatonin Restores Muc2 Depletion Induced by V. vulnificus VvpM via melatonin receptor 2 coupling with Gαq. J Biomed Sci (2020) 27:21. 10.1186/s12929-019-0606-x 31906951 PMC6943958

[201] Debnath B Islam W Li M Sun Y Lu X Mitra S. Melatonin Mediates Enhancement of Stress Tolerance in Plants. Int J Mol Sci (2019) 20:1040. 10.3390/ijms20051040 PMC6429401 30818835

[202] Yin L Wang P Li M Ke X Li C Liang D. Exogenous Melatonin Improves Malus Resistance to Marssonina Apple Blotch. J Pineal Res (2013) 54:426–34. 10.1111/jpi.12038 23356947

[203] Mansfield J Genin S Magori S Citovsky V Sriariyanum M Ronald P. Top 10 Plant Pathogenic Bacteria in Molecular Plant Pathology. Mol Plant Pathol (2012) 13:614–29. 10.1111/j.1364-3703.2012.00804.x PMC6638704 22672649

[204] Mahmood T Jan A Kakishima M Komatsu S. Proteomic Analysis of Bacterial-Blight Defense-Responsive Proteins in Rice Leaf Blades. Proteomics (2006) 6:6053–65. 10.1002/pmic.200600470 17051650

[205] Li C He Q Zhang F Yu J Li C Zhao T. Melatonin Enhances Cotton Immunity to Verticillium Wilt Via Manipulating Lignin and Gossypol Biosynthesis. Plant J (2019) 100:784–800. 10.1111/tpj.14477 31349367 PMC6899791

[206] Lee HY Byeon Y Tan DX Reiter RJ Back K. Arabidopsis Serotonin N-acetyltransferase Knockout Mutant Plants Exhibit Decreased Melatonin and Salicylic Acid Levels Resulting in Susceptibility to an Avirulent Pathogen. J Pineal Res (2015) 58:291–9. 10.1111/jpi.12214 25652756

[207] Zhao H Xu L Su T Jiang Y Hu L Ma F. Melatonin Regulates Carbohydrate Metabolism and Defenses Against Pseudomonas Syringae Pv. Tomato DC3000 Infection in Arabidopsis Thaliana. J Pineal Res (2015) 59:109–19. 10.1111/jpi.12245 25958775

[208] Qian Y Tan DX Reiter RJ Shi H. Comparative Metabolomic Analysis Highlights the Involvement of Sugars and Glycerol in Melatonin-Mediated Innate Immunity Against Bacterial Pathogen in Arabidopsis. Sci Rep (2015) 5:15815. 10.1038/srep15815 26508076 PMC4623600

[209] Shi H Chen Y Tan DX Reiter RJ Chan Z He C. Melatonin Induces Nitric Oxide and the Potential Mechanisms Relate to Innate Immunity Against Bacterial Pathogen Infection in Arabidopsis. J Pineal Res (2015) 59:102–8. 10.1111/jpi.12244 25960153

[210] Lee HY Back K. Mitogen-Activated Protein Kinase Pathways are Required for Melatonin-Mediated Defense Responses in Plants. J Pineal Res (2016) 60:327–35. 10.1111/jpi.12314 26927635

[211] Lee HY Back K. Melatonin is Required for H2 O2 - and NO-Mediated Defense Signaling Through MAPKKK3 and OXI1 in Arabidopsis Thaliana. J Pineal Res (2017) 62:e12379. 10.1111/jpi.12379 27862280

[212] Wurtman RJ Lieberman HR. Melatonin Secretion as a Mediator of Circadian Variations in Sleep and Sleepiness. J Pineal Res (1985) 2:301–3. 10.1111/j.1600-079X.1985.tb00647.x 3831313

[213] Li Y Ma J Yao K Su W Tan B Wu X. Circadian Rhythms and Obesity: Timekeeping Governs Lipid Metabolism. J Pineal Res (2020) 69:e12682. 10.1111/jpi.12682 32656907

[214] Fathizadeh H Mirzaei H Asemi Z. Melatonin: An Anti-Tumor Agent for Osteosarcoma. Cancer Cell Int (2019) 19:319. 10.1186/s12935-019-1044-2 31798348 PMC6884844

[215] Nabavi SM Nabavi SF Sureda A Xiao J Dehpour AR Shirooie S. Anti-Inflammatory Effects of Melatonin: A Mechanistic Review. Crit Rev Food Sci Nutr (2019) 59:S4–s16. 10.1080/10408398.2018.1487927 29902071

[216] Paulose JK Cassone VM. The Melatonin-Sensitive Circadian Clock of the Enteric Bacterium Enterobacter Aerogenes. Gut Microbes (2016) 7:424–7. 10.1080/19490976.2016.1208892 PMC5154366 27387841

[217] Ren W Wang P Yan J Liu G Zeng B Hussain T. Melatonin Alleviates Weanling Stress in Mice: Involvement of Intestinal Microbiota. J Pineal Res (2018) 64:e12448. 10.1111/jpi.12448 28875556

[218] Junaid A Tang H van Reeuwijk A Abouleila Y Wuelfroth P van Duinen V. Ebola Hemorrhagic Shock Syndrome-on-a-Chip. iScience (2020) 23:100765. 10.1016/j.isci.2019.100765 31887664 PMC6941864

[219] Bonilla E Valero-Fuenmayor N Pons H Chacín-Bonilla L. Melatonin Protects Mice Infected With Venezuelan Equine Encephalomyelitis Virus. Cell Mol Life Sci (1997) 53:430–4. 10.1007/s000180050051 PMC11147212 9176561

[220] Montiel M Bonilla E Valero N Mosquera J Espina LM Quiroz Y. Melatonin Decreases Brain Apoptosis, Oxidative Stress, and CD200 Expression and Increased Survival Rate in Mice Infected by Venezuelan Equine Encephalitis Virus. Antivir Chem Chemother (2015) 24:99–108. 10.1177/2040206616660851 27503577 PMC5890526

[221] Crespo I San-Miguel B Sánchez DI González-Fernández B Álvarez M González-Gallego J. Melatonin Inhibits the Sphingosine Kinase 1/sphingosine-1-phosphate Signaling Pathway in Rabbits With Fulminant Hepatitis of Viral Origin. J Pineal Res (2016) 61:168–76. 10.1111/jpi.12335 27101794

[222] Huang SH Cao XJ Liu W Shi XY Wei W. Inhibitory Effect of Melatonin on Lung Oxidative Stress Induced by Respiratory Syncytial Virus Infection in Mice. J Pineal Res (2010) 48:109–16. 10.1111/j.1600-079X.2009.00733.x 20070490

[223] Ellis LC. Melatonin Reduces Mortality From Aleutian Disease in Mink (Mustela Vison). J Pineal Res (1996) 21:214–7. 10.1111/j.1600-079x.1996.tb00288.x 8989719

[224] Huang SH Liao CL Chen SJ Shi LG Lin GJ. Melatonin Possesses an Anti-Influenza Potential Through its Immune Modulatory Effect. J Funct Foods (2019) 58:189–98. 10.1016/j.jff.2019.04.062

[225] Shneider A Kudriavtsev A. Can Melatonin Reduce the Severity of COVID-19 Pandemic? Int Rev Immunol (2020) 39:153–62. 10.1080/08830185.2020.1756284 32347747

[226] Zhou Y Hou Y Shen J Huang Y Martin W. Network-Based Drug Repurposing for Novel Coronavirus 2019-Ncov/SARS-Cov-2. Cell Discov (2020) 6:14. 10.1038/s41421-020-0153-3 PMC7073332 32194980

[227] Bahrampour Juybari K Pourhanifeh MH Hosseinzadeh A Hemati K Mehrzadi S. Melatonin Potentials Against Viral Infections Including COVID-19: Current Evidence and New Findings. Virus Res (2020) 287:198108. 10.1016/j.virusres.2020.198108 32768490 PMC7405774

